# Response to Intravenous Allogeneic Equine Cord Blood-Derived Mesenchymal Stromal Cells Administered from Chilled or Frozen State in Serum and Protein-Free Media

**DOI:** 10.3389/fvets.2016.00056

**Published:** 2016-07-22

**Authors:** Lynn B. Williams, Carmon Co, Judith B. Koenig, Crystal Tse, Emily Lindsay, Thomas G. Koch

**Affiliations:** ^1^Department of Biomedical Sciences, University of Guelph, Guelph, ON, Canada; ^2^Department of Clinical Studies, Ontario Veterinary College, University of Guelph, Guelph, ON, Canada

**Keywords:** horse, equine, stem cell transport, hypothermic, cryopreservation

## Abstract

Equine mesenchymal stromal cells (MSC) are commonly transported, chilled or frozen, to veterinary clinics. These MSC must remain viable and minimally affected by culture, transport, or injection processes. The safety of two carrier solutions developed for optimal viability and excipient use were evaluated in ponies, with and without allogeneic cord blood-derived (CB) MSC. We hypothesized that neither the carrier solutions nor CB-MSC would elicit measurable changes in clinical, hematological, or biochemical parameters. In nine ponies (study 1), a bolus of HypoThermosol^®^ FRS (HTS-FRS), CryoStor^®^ CS10 (CS10), or saline was injected IV (*n* = 3/treatment). Study 2, following a 1-week washout period, 5 × 10^7^ pooled allogeneic CB-MSCs were administered IV in HTS-FRS following 24 h simulated chilled transport. Study 3, following another 1-week washout period 5 × 10^7^ pooled allogeneic CB-MSCs were administered IV in CS10 immediately after thawing. Nine ponies received CB-MSCs in study 2 and 3, and three ponies received the cell carrier media without cells. CB-MSCs were pooled in equal numbers from five unrelated donors. In all studies, ponies were monitored with physical examination, and blood collection for 7 days following injection. CD4 and CD8 lymphocyte populations were also evaluated in each blood sample. In all three studies, physical exam, complete blood cell count, serum biochemistry, and coagulation panel did not deviate from established normal ranges. Proportions of CD4^+^ and CD8^+^ lymphocytes increased at 168 h postinjection in CB-MSC treatment groups regardless of the carrier solution. Decreases in CD4^+/^CD8^+^ double positive populations were observed at 24 and 72 h in CB-MSC-treated animals. There was no difference in viability between CB-MSCs suspended in HTS-FRS and CS10. HTS-FRS and CS10 used for low volume excipient injection of MSC suspensions were not associated with short-term adverse reactions. HTS-FRS and CS10 both adequately maintain CB-MSC viability following hypothermic or frozen simulated transport, respectively. CB-MSCs do not elicit clinical abnormalities, but allogeneic stimulation of CD4^+^ and CD8^+^ lymphocyte populations may occur. Future studies should include *in vitro or in vivo* evaluation of cell-mediated or adaptive immunity to autologous, identical allogeneic, or MSC originating from additional unrelated individuals in order to better characterize this response.

## Introduction

Equine mesenchymal stromal cells (MSCs) have been isolated from a variety of tissues including bone marrow (1–3), adipose tissue (3, 4), umbilical cord blood (CB) (5, 6), and umbilical cord tissue (3, 7). Collection of CB is a non-invasive source of CB-MSCs, which are highly proliferative, capable of trilineage differentiation, and possess lymphocyte suppressive properties in mixed lymphocyte reactions (5, 6, 8–10).

Currently in veterinary medicine, MSC preparations are available following a period of *ex vivo* culture expansion for autologous administration. Such use is limited by the several weeks needed for preparation of cultured MSC from autologous cell or tissue samples, effectively excluding immediate treatment of acute injuries. Allogeneic MSC use allows treatment of acute lesions with prescreened and characterized MSC cultures. In fact, the future of regenerative medicine may include cryopreserved MSC suspensions that have been screened for desirable characteristics being stocked at veterinary primary care facilities for immediate treatment at the time of diagnosis of injury.

Allogeneic MSC use has been shown to be safe for *in vivo* intravenous (11, 12), intraarticular (13–16), intradermal (17), intrathecal (18), and intralesional-tendon injection in the horse (19–21). Although there are reports of an increased inflammatory response following intraarticular injection of allogeneic MSC (14), inflammation subsided quickly and no difference in immune response was detected between allogeneic and autologous MSC (22).

Regardless of MSC source or allogeneic/autologous use, MSC suspensions require transport from laboratory to a veterinary clinic, usually by commercial overnight carrier. Decreased MSC viability has been demonstrated following transportation in various media (23, 24). One strategy reported to slow the decrease in MSC viability is to transport MSC in serum-containing media. If the serum is allogeneic or xenogeneic in nature, then it is often recommended that the MSCs undergo multiple washes prior to injection to remove the majority of foreign antigens introduced by the serum. This process is inconvenient, associated with a decrease of total MSC numbers and increases the risk of bacterial contamination. A cell carrier media that requires removal as described, prior to injection of the MSC, is referred to as an ancillary media. Excipient media is a cell-carrier solution that provides necessary support but is otherwise unreactive and can be injected with the MSC. Excipient media allows a convenient and more standardized final product formulation since no manipulations are required at the time of treatment. HypoThermosol^®^ FRS (HTS-FRS, BioLife Solutions, Bothell, WA, USA) and CryoStor^®^ (CS, BioLife Solutions, Bothell, WA, USA) are cell preservation media for excipient use with chilled or frozen mammalian cells, respectively. These media are serum free and protein free, commercially available, procured with adherence to current Good Manufacturing Practices (GMP) and are optimized for maintaining cell viability at 2–8°C or in a frozen state. HTS-FRS has been shown to preserve viability of cells, tissues, or organs better than cells, tissues, or organs transported in many other media (25–30). These media mimic intracellular solute concentrations, which minimize solute gradients across cell membranes providing an optimal environment for hypothermic or cryogenic (DMSO added) storage (29, 31). CryoStor CS10 is a cryomedia pre-formulated with 10% DMSO. In our lab, improved viability over non-serum-containing media and equivalence to serum-containing media has been demonstrated in preservation of equine MSC chilled in HTS-FRS or frozen in CS10 (see File S1 in Supplementary Material). Additionally, our research group has compared HTS-FRS to phosphate buffered saline (PBS) for administration by intra-articular injection. A similarly mild, clinically insignificant inflammatory reaction was observed following intra-articular injection of both PBS and HTS-FRS (see File S2 in Supplementary Material).

We hypothesized that the carrier solutions alone or in combination with CB-MSC would not elicit measurable changes in clinical parameters, hematological including CD4^+^ and CD8^+^ counts and serum biochemical parameters, in healthy ponies.

## Materials and Methods

### Collection, Isolation, and Culture of CB-MSC

Cryopreserved CB-MSC cultures from frozen stock were used. CB-MSCs were procured as previously described (5, 6) Previously, CB-MSCs isolated under identical conditions displayed a consistent phenotype before and after cryopreservation. This included high expression of CD29, CD44, CD90, and absent or low expression of major histocompatibility complex (MHC) class I, MHC-II, CD4, CD8, CD11a/18, and CD73 (10). CB-MSC cultures from five unrelated donor foals were expanded in culture to achieve necessary MSC numbers in DMEM-containing 30% FBS, 1% Penicillin/Streptomycin, and 1% l-glutamine. CB-MSC cultures suspended in HTS-FRS had been cryopreserved once, were between passage 6 and passage 7, and had been cultured for a total of 42–50 days. CB-MSC cultures suspended in CS10 had been cryopreserved once, were between passage 4 and passage 5, and had been cultured for a total of 35–40 days. Twenty-four hours prior to injection, MSC suspended in HTS-FRS were detached from cell culture flasks using trypsin EDTA, washed once in PBS, then suspended in HT-FRS and stored in a reusable temperature-controlled shipping container (Greenbox 2–8°C thermal management system, ThermoSafe^®^, Arlington Heights, IL, USA) in the lab until just prior to the time of injection. MSCs suspended in CS10 were detached from cell culture flasks 18 days prior to injection, gradually frozen at a rate of −1°C/min to −80°C before transfer to liquid nitrogen storage. These CB-MSCs were transported to the research farm on dry ice and thawed under lukewarm tap water approximately 20 min prior to injection. Acellular vials of HTS-FRS or CS10 (to act as controls) were handled in an identical manner to their respective CB-MSC containing vials.

### Research Animals

Twelve mature healthy ponies (4 females, 8 males; age 4 years) that had not received any medications for at least 2 months were used in these studies. Physical exam and baseline CBC, biochemistry, and coagulation profiles indicated all horses were free from obvious disease. All procedures complied with institutional animal care committee protocols approved for this study (Koch, University of Guelph Animal Care Protocol #3247).

### Experimental Protocol

#### Study 1

Ponies were randomly assigned into treatment groups by lottery. Prior to injection, baseline (0 h) physical examination was performed including temperature, pulse, respiration, and demeanor, and 12 mL blood was collected from the jugular vein. A single 10 mL injection of one of CS10 (*n* = 3), HTS-FRS (*n* = 3), or physiologic saline (*n* = 3) was administered to nine ponies into the jugular vein. The remaining three ponies were not used in study 1. Temperature, pulse, and respiration were recorded from all treated ponies at 0, 1, 3, 6, 12, 24, 48, 72, 168 h postinjection. About 12 mL of whole blood was collected from treated ponies at 0, 1, 24, 72, and 168 h postinjection.

#### Study 2

Ponies were randomly assigned into treatment groups as indicated above. All 12 ponies received a single IV injection of either 10 mL HTS-FRS (*n* = 3) or 5 × 10^7^ pooled allogeneic CB-MSC (1 × 10^7^ CB-MSCs from five unrelated MSC donors) (*n* = 9). Ponies were monitored, and blood samples collected as indicated above.

#### Study 3

Ponies were once again randomly assigned into treatment groups as indicated above. All 12 ponies received a single IV injection of either 10 mL CS10 (*n* = 3) or 5 × 10^7^ pooled allogeneic CB-MSCs (1 × 10^7^ CB-MSC from five unrelated MSC donors) suspended in CS10 (*n* = 9). Ponies were monitored and blood samples collected as indicated above.

### Evaluation of Post-Transport Viability

Following injection, vials that had been used to transport CB-MSC suspensions were transported back to the laboratory at room temperature where MSC viability was calculated using the residual ~100–200 μL of CB-MSC suspension in each vial using a hemocytometer counting chamber and the trypan blue exclusion assay. In total, CB-MSC suspensions had been stored under transport conditions (2–8°C in study 2 and dry ice cooler in study 3) for 24 h followed by approximately 2 h at room temperature before viability measurements were obtained.

### Evaluation of Blood Samples

Blood samples were submitted to our veterinary diagnostic laboratory for complete blood count (Avida 2120i, Siemens Canada, Oakville, ON, USA), serum biochemistry profile, and coagulation profile (PT, PTT, fibrinogen). In addition, CD4^+^ and CD8^+^ lymphocyte populations were analyzed by flow cytometry. Briefly, whole blood was prepared by combining 100 μL of whole blood with 1400 μL 1× RBC lysis buffer (9 parts dH_2_O, 1 part 10× RBC lysis buffer (1.7M NH_4_Cl, 0.1M KHCO_3_, 1 mM NH_4_EDTA, pH 7.3)) and incubated 5 min at room temperature. About 1 mL flow buffer (PBS/1% horse serum, 15 mM Na azide, 0.5 mM NA_4_EDTA) was added and spun at 1200 rpm for 5 min. Separate 15 min incubations followed with CD4 and then CD8 antibodies (catalog # MCA108PE & MCA1078F, AbD Serotec, Raleigh, NC, USA) with the cell pellet washed in flow buffer after each incubation. Finally, the cell pellet was suspended in flow buffer for flow cytometry (BD Accuri C6, BD Biosciences, Mississauga, ON, USA). Flow cytometry gates were set to first select a distinct cell population with size and granularity consistent with equine lymphocytes. From this population 10,000 cells were evaluated for CD4^±^ and CD8^±^ surface markers. Gates to differentiate cell populations were maintained consistent throughout the experiment.

### Statistical Analysis

Raw data from physical exams, CBC, biochemistry profiles, and coagulation profiles were imported into statistical software (SAS 9.2, SAS institute, Carey, NC, USA). Data were randomly blocked by horse and were in the form of a split plot in time design; hence, there were repeated measures in time. Data were analyzed using a general linear mixed model to evaluate fixed effects (treatment, time and treatment by time interaction) and random effects (horse) using the PROC MIXED function. Various error structures, supplied by the statistical analysis software (AR, ARH, TOEP, banded TOEP 2-TOEP (t-1), TOEPH, banded TOEPH2-TOEPH t-1, unstructured, banded unstructured 2-banded unstructured t-1) were tested, and one error structure was selected for each model based on the lowest Akaike information criterion (AIC). To improve the power of each study identical treatments were combined between studies 1–2 (HTS-FRS) and studies 1–3 (CS10) after determining there was no study effect in the linear model. Residual analysis was performed on each dataset to determine if ANOVA assumptions were met, detect potential outliers, and evaluate the need for data transformation. The residuals were formally tested for normality using the four tests offered by SAS (Shapiro-Wilk, Kolmogorov–Smirnov, Cramer-von Mises, Anderson-Darling) and plotted against the predicted values and explanatory variables used in the model. For the purpose of determining statistical significance, α was set at 0.05.

Data resulting from postinjection CB-MSC viability measurements were analyzed in a similar manner using the panel of tests for normality and methodology for selecting an appropriate error structure as indicated above. Following this analysis, a general linear model and the PROC MIXED function (SAS 9.2, SAS Institute, Cary, NC, USA) were performed to evaluate solution effects on MSC viability.

## Results

Following statistical analysis, data were standardized by dividing by the pretreatment mean. This allowed the many values resulting from the CBC, biochemistry profile, and coagulation panel to be plotted on the same scale.

**Figure 1:**
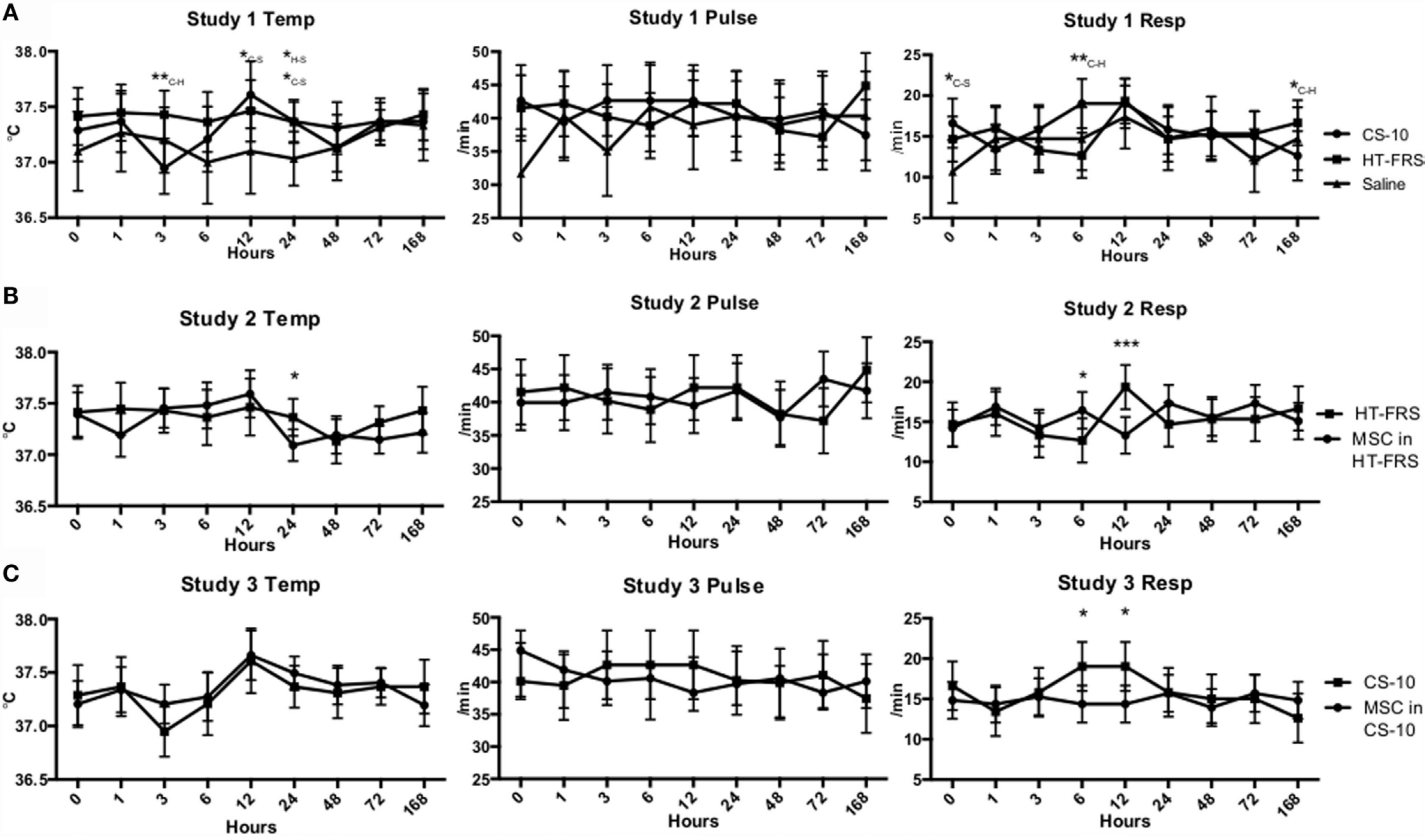
**(A)** Temperature, pulse, and respiration rates of ponies receiving a 10 mL IV bolus of HypoThermosol^®^ FRS (HTS-FRS) (*n* = 6), CryoStor^®^ CS10 (CS10) (*n* = 6), or saline (*n* = 3). **(B)** Temperature, pulse, and respiration rates of ponies receiving an IV injection of 5 × 10^7^ pooled allogeneic umbilical cord blood derived mesenchymal stromal cells (CB-MSC) suspended in 10 mL HTS-FRS (*n* = 9) or 10 mL HTS-FRS alone (*n* = 6). **(C)** Temperature, pulse, and respiration rates of ponies receiving an IV injection of 5 × 10^7^ pooled allogeneic umbilical cord blood derived mesenchymal stromal cells (CB-MSC) suspended in 10 mL CS10 (*n* = 9) or 10 mL CS10 alone (*n* = 6). Data points indicate mean response with error bars indicating 95% CI. *p < 0.05, **p < 0.01, ***p < 0.001 compared to the corresponding time point. In study 1, subscript letters specifies the treatments that the difference occurs between, C-CS10, H-HTS-FRS, S-Saline.

Physical exam parameters (temperature, pulse rate, and respiration rate) remained within normal limits throughout the duration of all three studies (Figure 1). Differences in temperature and respiratory rate were observed as indicated in Figure 1. Throughout the duration of the study, all animals remained bright, alert, and responsive.

**Figure 2:**
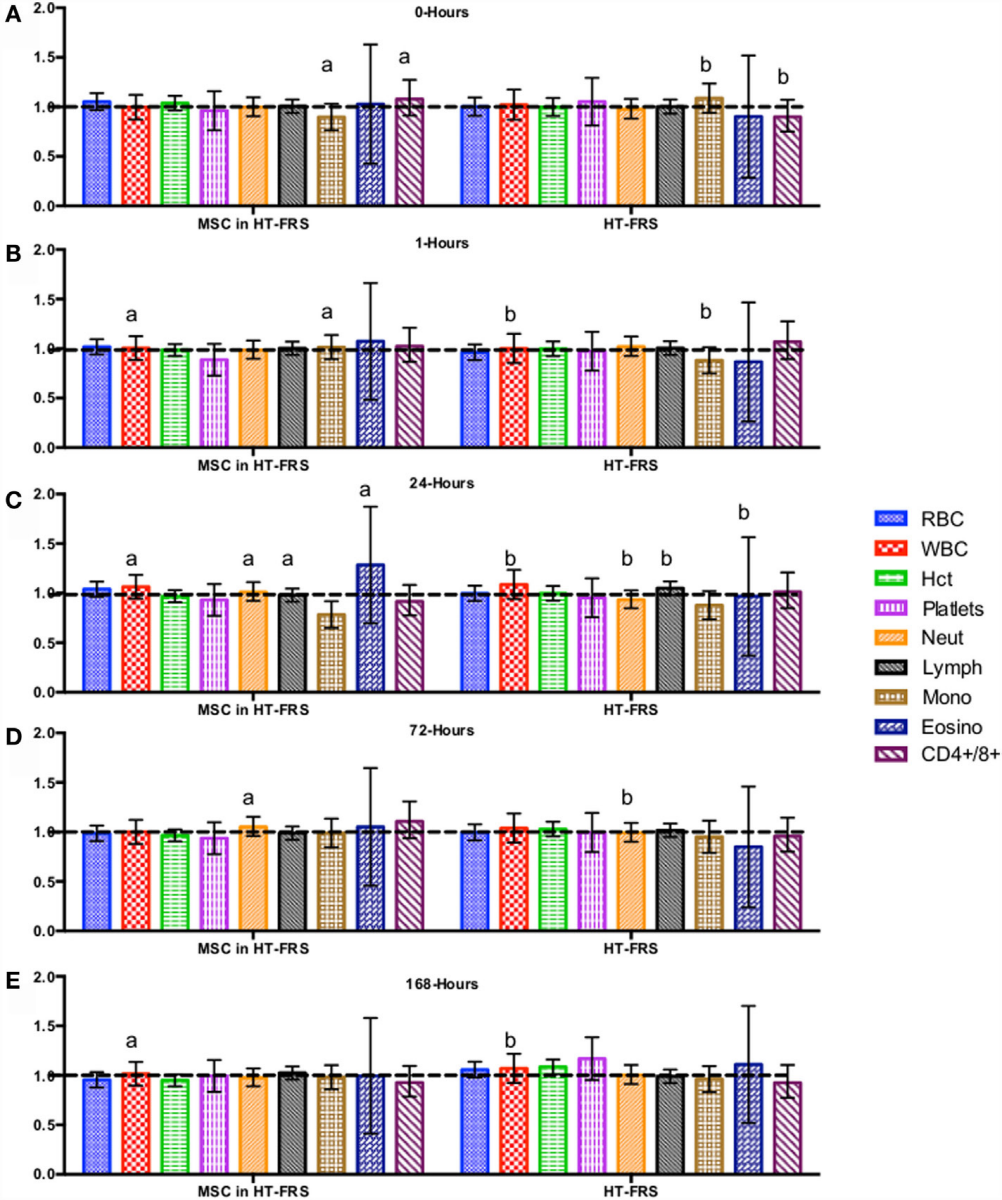
**Percent difference from pre-injection measurements (black dotted line) of complete blood count (A) 0 h, (B) 1 h, (C) 24 h (D) 72 h, (E) 168 h following intravenous injection of 5 × 10^7^ pooled allogeneic CB-MSC derived from five horses**. 10 mL HypoThermosol^®^ (HTS-FRS) control. Error bars represent 95% confidence interval. Different letters indicate statistical differences between treatment and control groups for a specific parameter.

**Figure 3:**
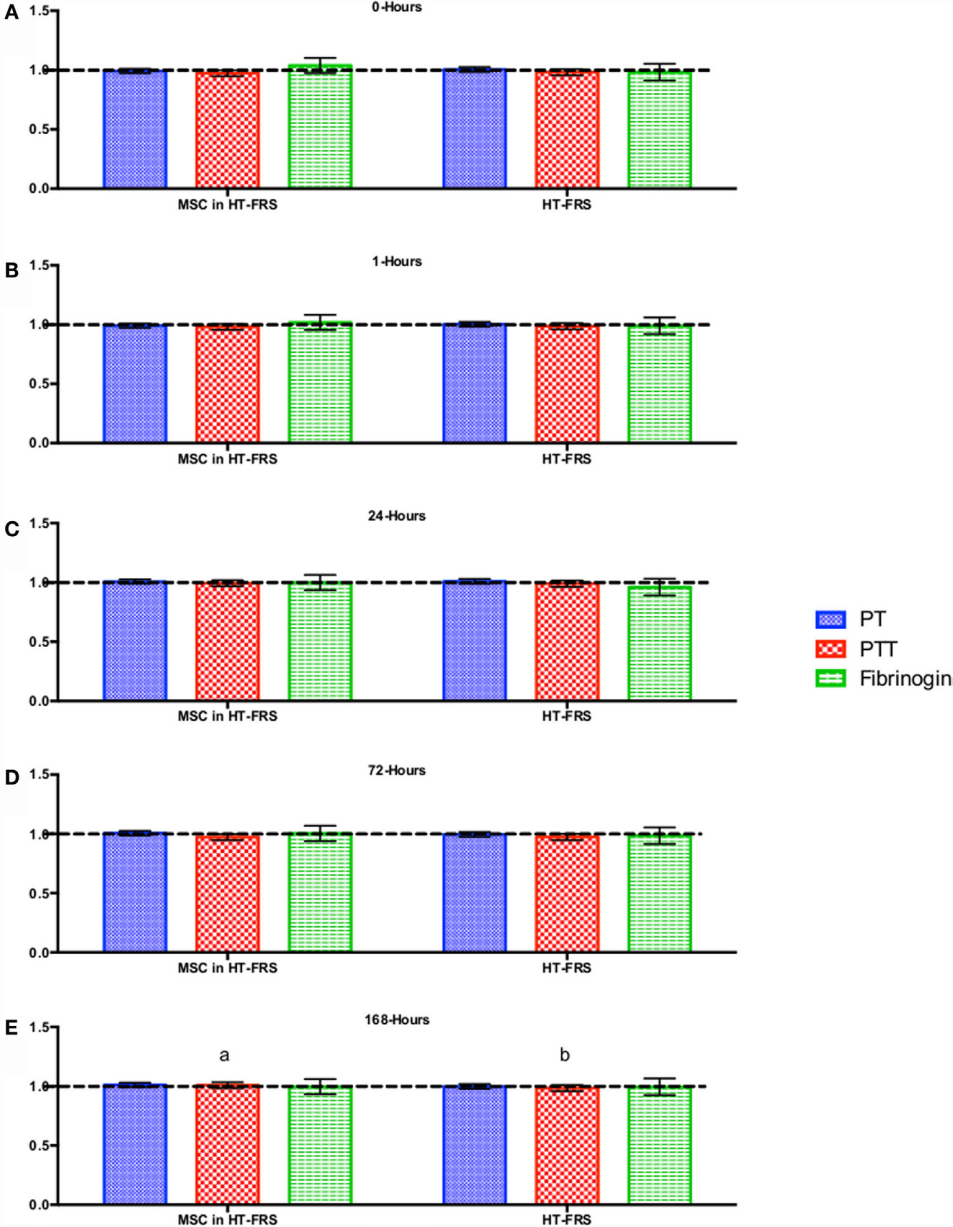
**Percent difference from pre-injection measurements (black dotted line) of coagulation profile (A) 0 h, (B) 1 h, (C) 24 h (D) 72 h, (E) 168 h following intravenous injection of 5 × 10^7^ pooled allogeneic CB-MSC derived from five horses**. 10 mL HypoThermosol^®^ (HTS-FRS) control. Error bars represent 95% confidence interval. Different letters indicate statistical differences between treatment and control groups for a specific parameter.

**Figure 4:**
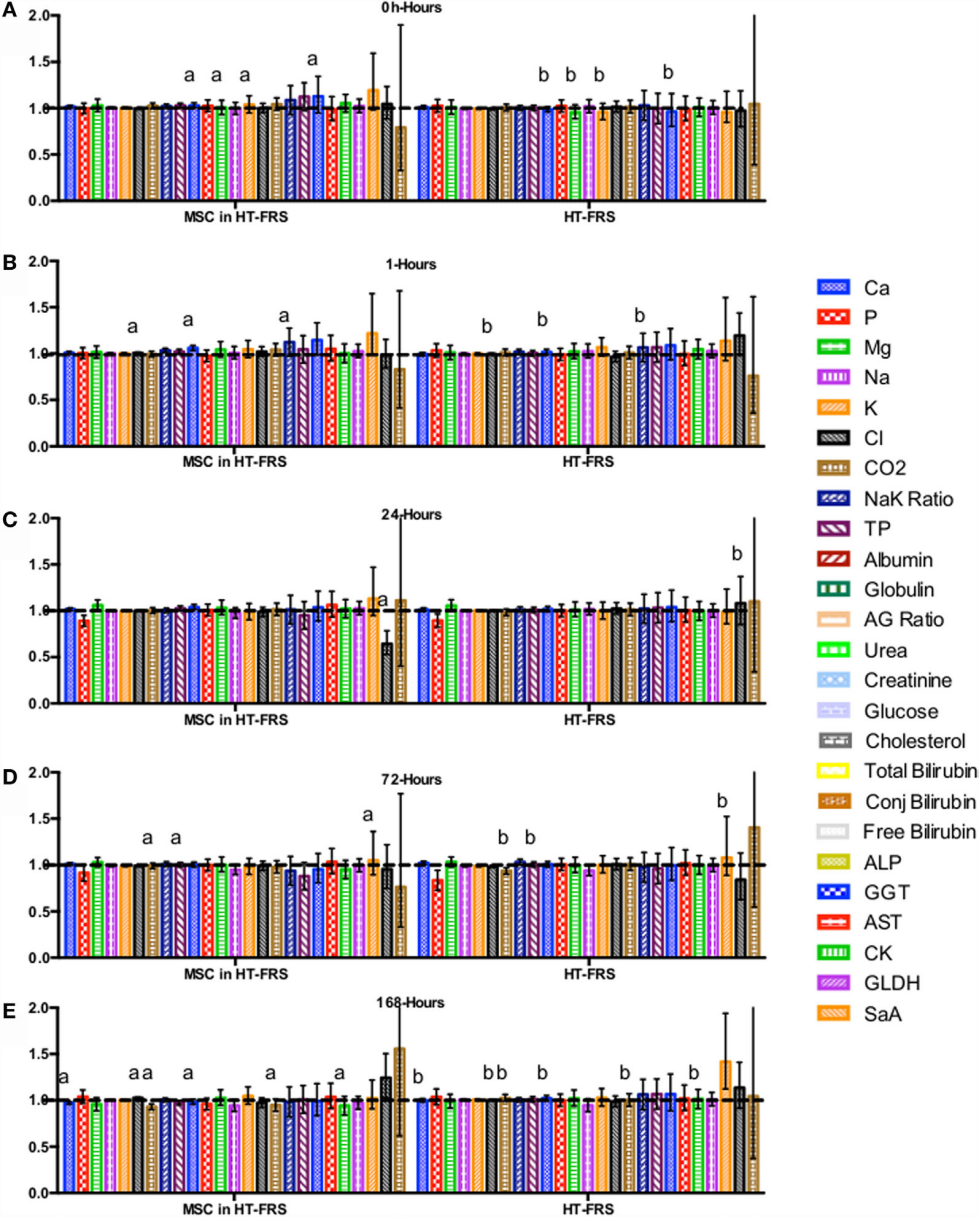
**Percent difference from pre-injection measurements (black dotted line) of biochemistry profile (A) 0 h, (B) 1 h, (C) 24 h (D) 72 h, (E) 168 h following intravenous injection of 5 × 10^7^ pooled allogeneic CB-MSC derived from five horses**. 10 mL HypoThermosol^®^ (HTS-FRS) control. Error bars represent 95% confidence interval. Different letters indicate statistical differences between treatment and control groups for a specific parameter.

**Figure 5:**
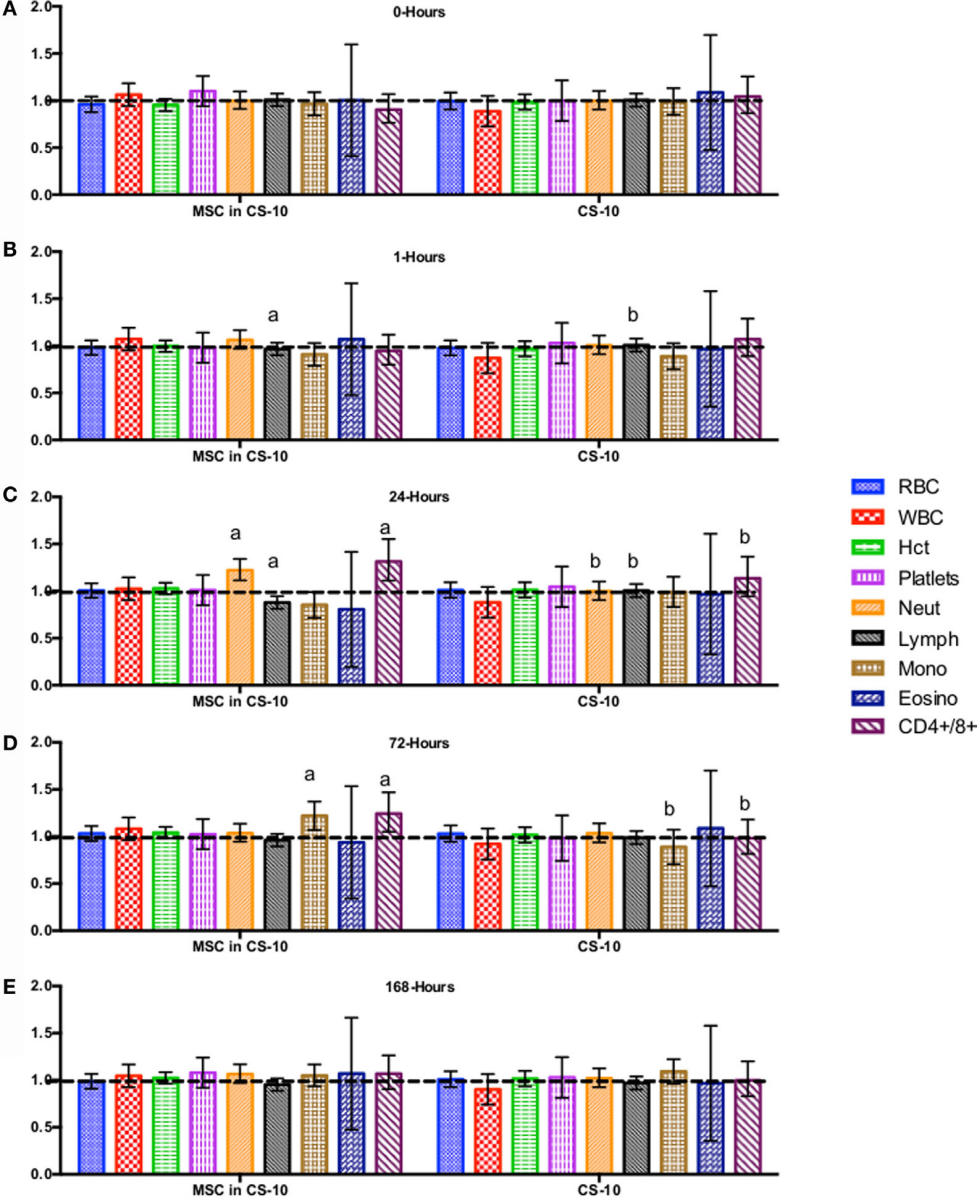
**Percent difference from pre-injection measurements (black dotted line) of complete blood count (A) 0 h, (B) 1 h, (C) 24 h (D) 72 h, (E) 168 h following intravenous injection of 5 × 10^7^ pooled allogeneic CB-MSC derived from 5 horses**. 10 mL CryoStor^®^ CS10 (CS10) control. Error bars represent 95% confidence interval. Different letters indicate statistical differences between treatment and control groups for a specific parameter.

**Figure 6:**
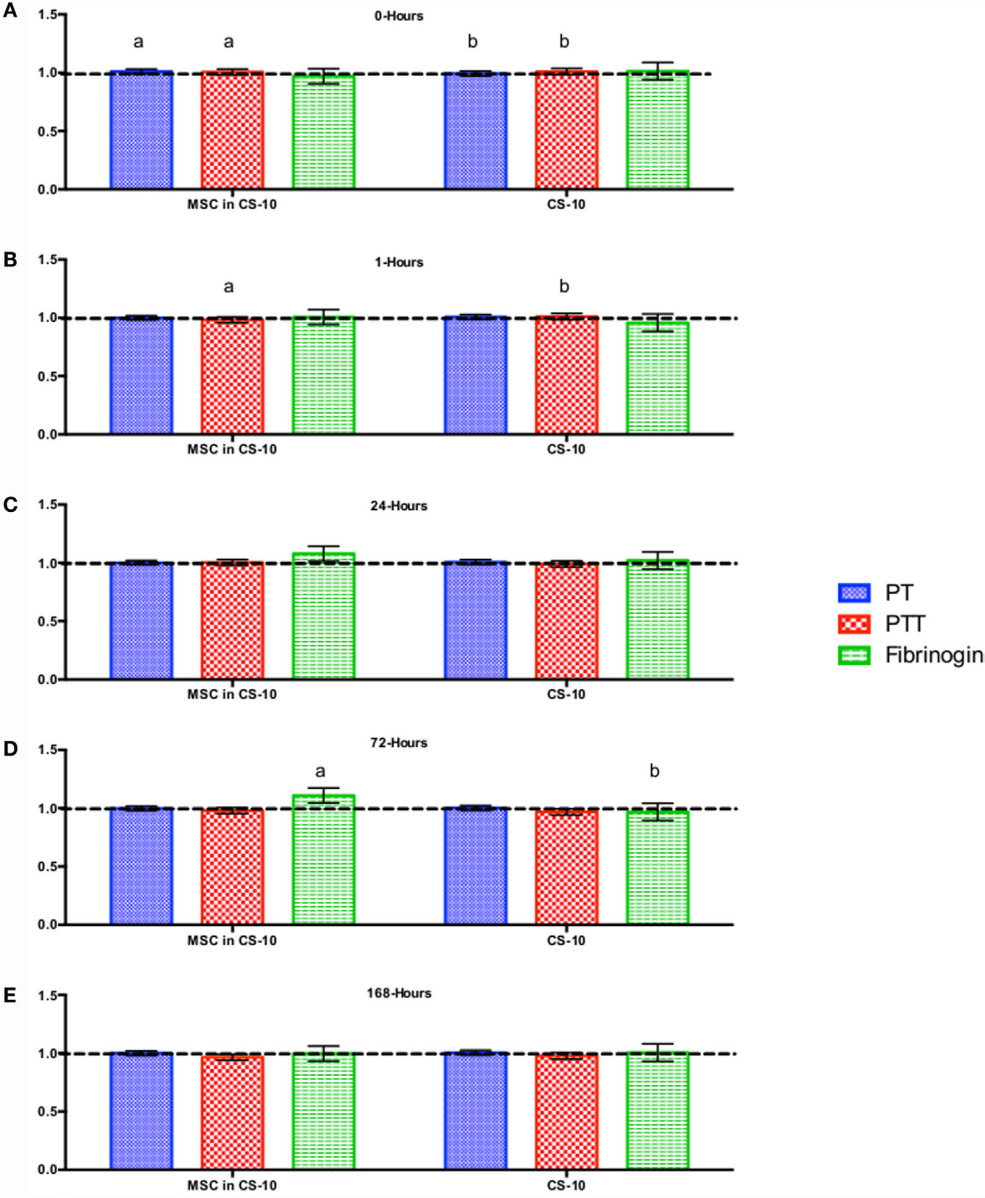
**Percent difference from pre-injection measurements (black dotted line) of coagulation profile (A) 0 h, (B) 1 h, (C) 24 h, (D) 72 h, and (E) 168 h following intravenous injection of 5 × 10^7^ pooled allogeneic CB-MSC derived from 5 horses**. 10 mL CryoStor^®^ CS10 (CS10) control. Error bars represent 95% confidence interval. Different letters indicate statistical differences between treatment and control groups for a specific parameter.

**Figure 7:**
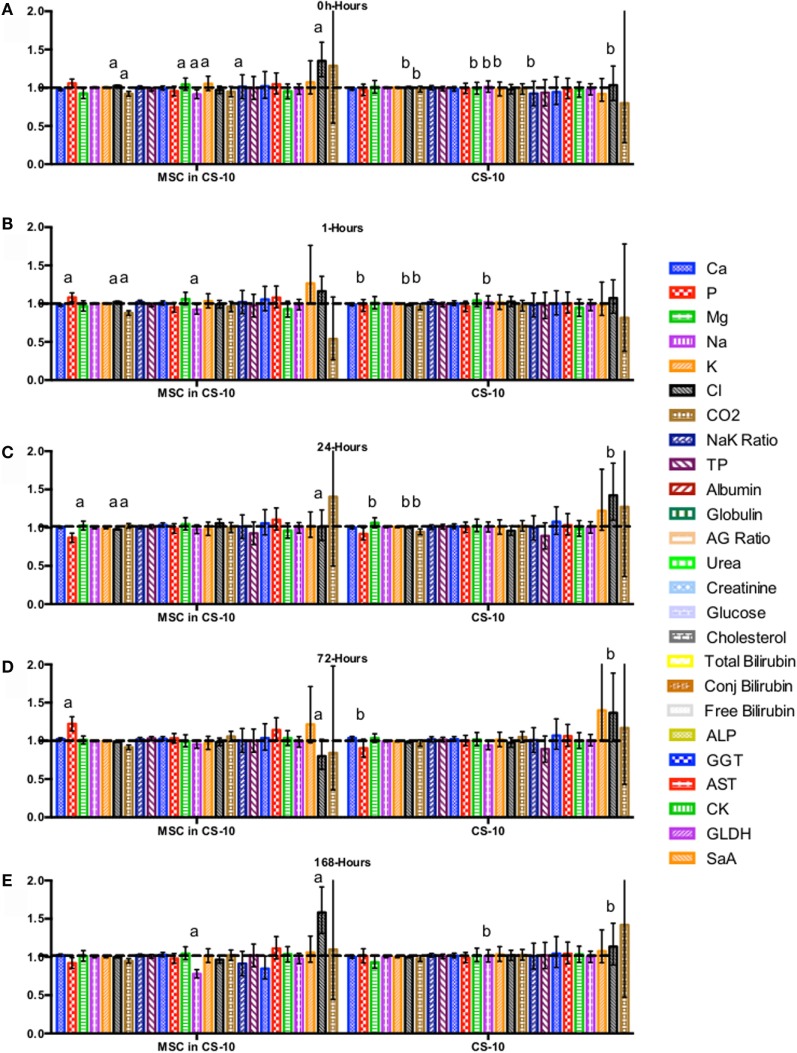
**Percent difference from pre-injection measurements (black dotted line) of biochemistry profile (A) 0 h, (B) 1 h, (C) 24 h (D) 72 h, (E) 168 h following intravenous injection of 5 × 10^7^ pooled allogeneic CB-MSC derived from 5 horses**. CryoStor^®^ CS10 (CS10) control. Error bars represent 95% confidence interval. Different letters indicate statistical differences between treatment and control groups for a specific parameter.

CBC, biochemistry profile, and coagulation profiles did not deviate from the normal ranges provided by the diagnostic laboratory. Significant differences in parameters are indicated in File S3 in Supplementary Material (for study 1) and Figures 2–7 for studies 2 and 3.

**Figure 8:**
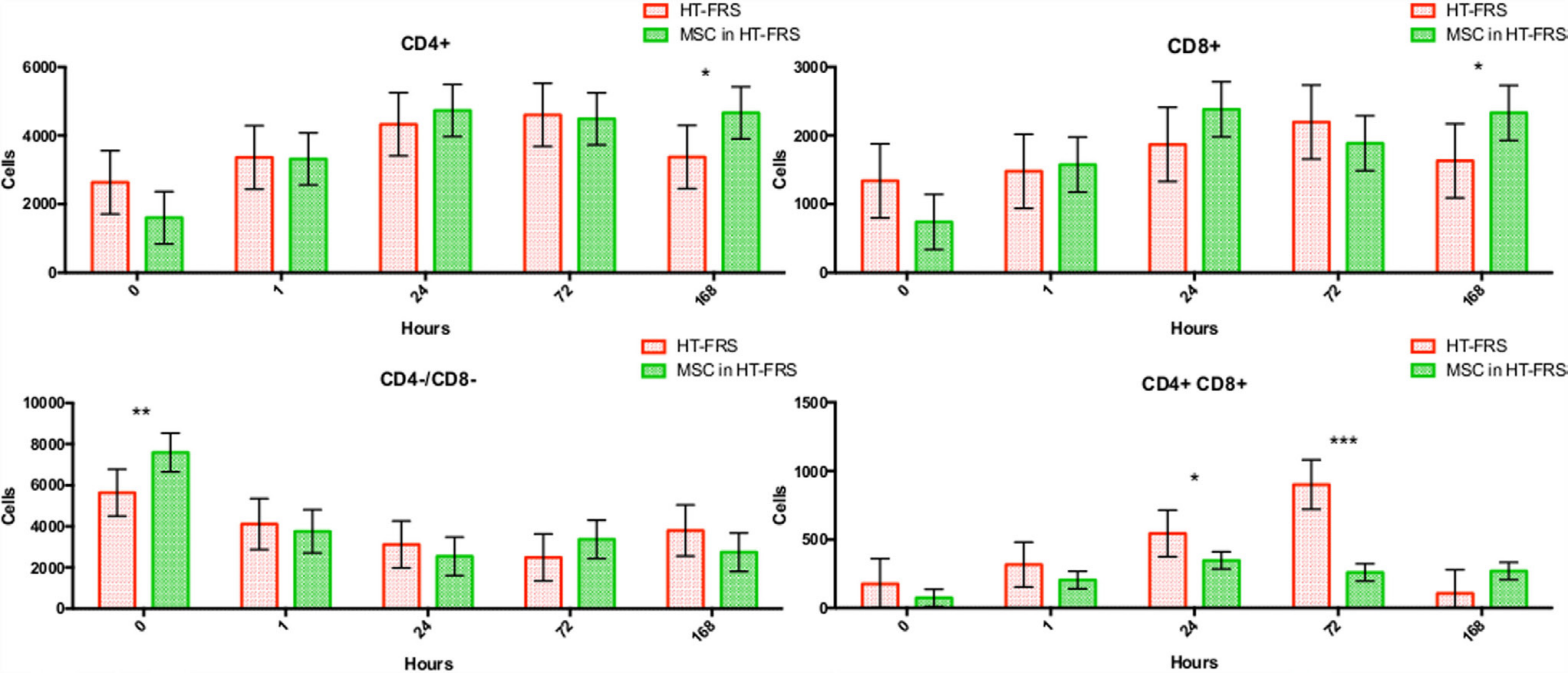
**CD4^+^, CD8^+^, CD4^+^/CD8^+^ (double positive), and CD4^−^/CD8^−^ (double negative) lymphocyte populations following intravenous injection of 5 × 10^7^ pooled allogeneic CB-MSC derived from five horses**. HypoThermosol^®^ FRS (HTS-FRS) control. *p < 0.05, **p < 0.01, ***p < 0.001.

**Figure 9:**
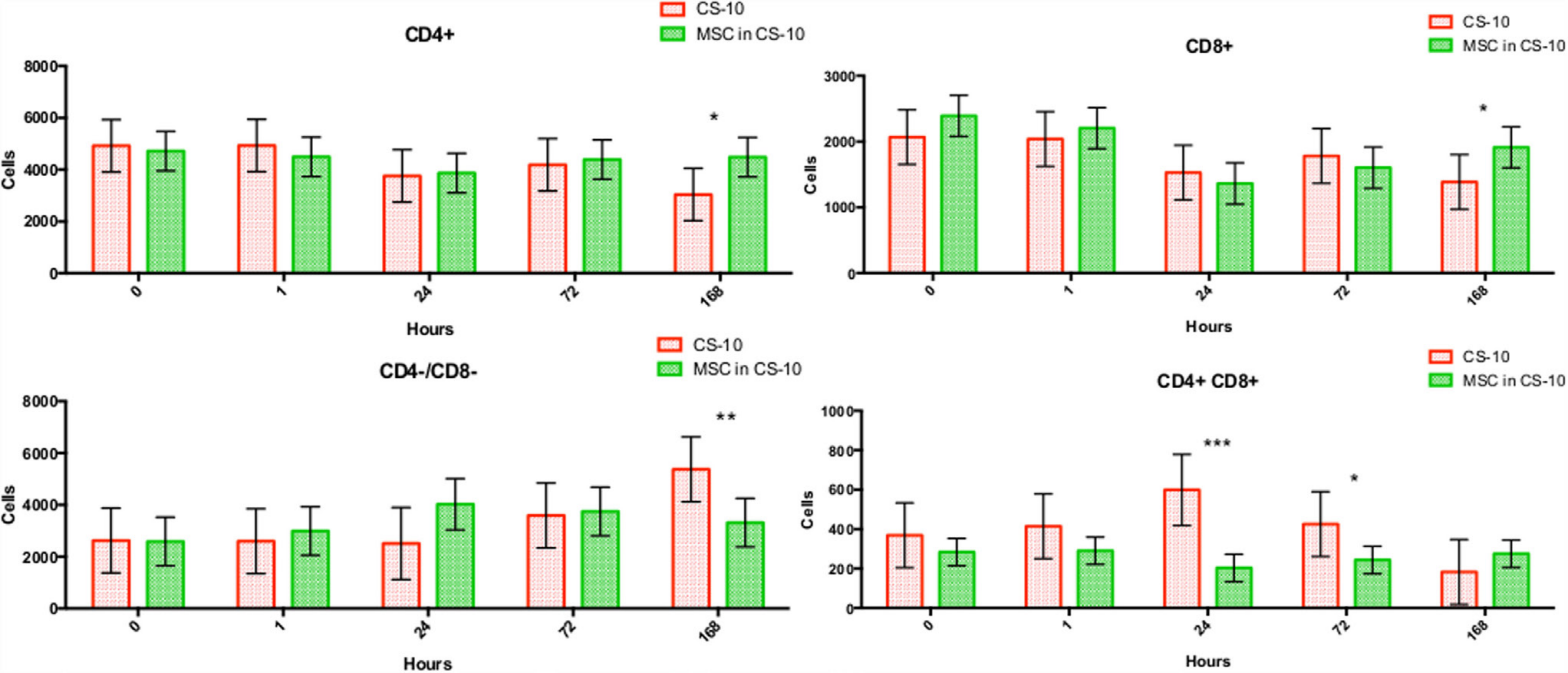
**CD4^+^, CD8^+^, CD4^+^/CD8^+^ (double positive), and CD4^−^/CD8^−^ (double negative) lymphocyte populations following intravenous injection of 5 × 10^7^ pooled allogeneic CB-MSC derived from five horses**. CryoStor^®^ CS10 (CS10) control. *p < 0.05, **p < 0.01, ***p < 0.001.

Flow cytometry of whole blood samples to assess CD4^+^ and CD8^+^ lymphocyte populations revealed a significant decrease in the double positive (CD4^+^, CD8^+^) population 24 and 72 h following MSC injection compared to matched HTS-FRS (*p* = 0.03, <0.0001, respectively) and CS10 (*p* < 0.0001, 0.04, respectively) controls. Significant elevation of CD4^+^ and CD8^+^ lymphocyte populations were observed at 168 h in animals receiving pooled allogeneic CB-MSC suspended in HTS-FRS [(CD4^+^: *p* = 0.03, CD8^+^: *p* = 0.03) and (CS10 CD4^+^: *p* = 0.02, CD8^+^: 0.03)]. The CD4:8 ratio was decreased at 72 h in CB-MSC in HTS-FRS-treated animals compared to HTS-FRS-treated animals alone (*p* = 0.04). The CD4:8 ratio was also decreased at 24 and 72 h in CS10 treated animals compared to CS10 treated animals alone (*p* = 0.04, 0.001, respectively, Figures 8 and 9).

Post injection the viability of residual CB-MSC suspensions was not different between CB-MSC suspended in HTS-FRS (mean-74.3, 95% CI: 69.5–78.5%) or CS10 (mean-76.0, 95% CI: 71.4–80.0%), *p* = 0.6, File S3-figure SE in Supplementary Material.

## Discussion

We report no adverse clinical reaction to the IV injection of 10 mL of either HTS-FRS or CS10 in healthy 4-year-old ponies. Although a number of statistical differences were detected in physical exam, CBC, biochemistry profile, or coagulation profile parameters, at no time were mean parameters outside of previously established normal ranges in a clinically significant manner. We consider these differences as minor variations from normal due to random variation within the herd and detection of differences as a result of chance. A similar lack of inflammatory response has been observed following IA injection of HTS-FRS in horses (see File S2 in Supplementary Material). Likewise, no adverse reactions were observed following IV injection of CB-MSC pooled from five unrelated horses. No abnormalities were detected in CBC, biochemistry, or coagulation profiles where mean values did not differ from established normal ranges. This indicates that although statistically different, physical examinations and routine blood work did not detect clinically significant abnormalities between treatment groups.

We did, however, observe differences in CD4^+^, CD8^+^, and double positive (both CD4^+^ and CD8^+^ receptors) lymphocyte populations. In general, we observed an increase in CD4^+^ and CD8^+^ populations at 168 h postinjection and a decrease in the double positive population at the 24 and 72 h time points as well (Figure 9). Increases in the CD4^+^ and CD8^+^ populations suggest allogeneic stimulation of both T-helper and Cytotoxic T-cell populations, respectively. While CD4^+^ and CD8^+^ lymphocytes are often considered mutually exclusive phenotypes and reportedly rare in human and dog (32, 33), they represent a high proportion of lymphocytes in swine and chickens (34–36). While our proportion of double positive lymphocytes was relatively small (less than 10%); lymphocytes originating from Thoroughbred horses and Connemara ponies have been reported to contain as high as 39% double positive lymphocytes (37). Although little is known about the function of this population, they are thought to appear or disappear from CD4^+^ lymphocytes through the gain or loss of the CD8^+^ receptor (37, 38). Interestingly, in the presence of MSC, CD4^+^/CD8^+^ double positive lymphocytes appear resistant to MSC-mediated lymphocyte suppression yet quickly disappear following a period of *in vitro* culture (37).

The viability reported following here following hypothermic storage or cryopreservation is considerably less than what we report under controlled conditions (File S1 in Supplementary Material) or that is reported by others using human BM-MSC (29). We attribute this difference to the time (~2 h) MSC samples spent at room temperature while injections were being performed and in transit back to the laboratory. Regardless of this, we report similar viability of MSC samples despite sample differences in the preservation method.

CB-MSCs isolated under similar conditions to those used in this study have consistently shown lymphocyte suppressive effects *in vitro* (10). These MSCs express CD29, CD44, CD90, and low/absent expression of MHC I, MHC II, CD4, CD8, CD11a/18, and CD73 markers (10). Despite a supposed lack of both MHC I and MHC II on these CB-MSCs, a significant shift of *in vivo* CD4^+^ and CD8^+^ lymphocyte populations may indicate a possible allogeneic reaction and a need to confirm the lack of MHCI/II markers of the CB-MSC populations injected. Additional research in these animals should include Fox3p staining to evaluate CD4^+^ regulatory T-lymphocytes (T_reg_) populations, administration of additional dose(s) of MSC with similar monitoring, or harvest of peripheral blood lymphocytes for evaluation for MSC-mediated lymphocyte suppressive properties. The immune response resulting from subsequent CB-MSC doses may reveal significant information as to the mechanisms by which allogeneic CB-MSCs elicit possible immune reaction. Numerous reports indicate allogeneic MSCs administered by various routes may prime the immune system for a greater response, following future MSC injections or organ transplant in the recipient animal, as reviewed by Griffin and colleagues (39). Such investigation may indicate formation of alloantibodies, increased CD8^+^ lymphocyte response, or the duration any potential immunity persists. This understanding would advance the understanding of potential ways allogeneic MSCs may be used to avoid stimulation of unwanted immune reactions. Although similar to horses in many ways the pony may be a more sensitive *in vivo* model for study of potentially immunogenic substances. Ponies express increased polymorphonucleocyte function and greater inflammatory response to inflammatory stimuli (40, 41). As a result, when evaluating a potentially immunogenic substance for safety the pony model may reveal responses that would not be observed in adult horses.

Although the makeup of these current proprietary solutions is not publicly available, a similar intracellular-like first generation base formulation has been published when “hypothermosol” was first described (42, 43). Assuming CryoStor and HTS-FRS are similar in intracellular-like components (aside from DMSO in CryoStor) (29, 31) the main safety concern for administration of these solutions is the high levels of K^+^ (42.5 meq/L) in solution. While IV administration of hyperkalemic solutions is contraindicated, excipient injection of cell suspensions in small volumes especially to large animal species eliminates risk of inducing cardiac conduction abnormalities following injection, since administration of 10 mL/kg/h (approaching 0.5 meq/kg/h maximum safe administration rate of K^+^) is very unlikely to occur when small volumes are used for regenerative medicine purposes.

In conclusion, HypoThermosol^®^ FRS (HTS-FRS) and CryoStor^®^ CS10 (CS10) used for low volume excipient injection of MSC suspensions was not associated with short-term adverse reactions. HTS-FRS and CS10 both adequately maintain CB-MSC viability following hypothermic or frozen simulated transport, respectively. CB-MSC does not elicit clinical abnormalities, but allogeneic stimulation of CD4^+^ and CD8^+^ lymphocyte populations may occur. Future studies should include *in vitro* or *in vivo* evaluation of cell-mediated or adaptive immunity to autologous, identical allogeneic, or MSC arising from additional unrelated individuals in order to more precisely determine the nature of this response.

## Author Contributions

LW contributed to study design, helped plan experiments, collected samples, was responsible for animal care, analyzed and interpreted data, drafted and edited this manuscript. CC, CT, and EL helped with *in vivo* data collection. JK provided materials, reagents, horses for the *in vivo* assessment of cells within synovial joints, helped with data collection, and interpreted data. TK designed the study and planned the experiments, provided reagents, materials, and laboratory space, performed IV injections, interpreted data, and edited the manuscript. All authors approved the final manuscript.

## Conflict of Interest Statement

TK acts in a volunteer capacity as non-executive Director, Scientific Affairs (ex officio) of eQcell therapies Inc., Aurora, ON, Canada, a company for which TK’s research laboratory provides equine stem cell isolation and storage services. TK holds a minor non-controlling share in eQcell therapies Inc. The remaining authors declare no competing interests.
