# Supplementary material for: Response to Intravenous Allogeneic Equine Cord Blood-Derived Mesenchymal Stromal Cells Administered from Chilled or Frozen State in Serum and Protein-Free Media
Source: Front Vet Sci. 2016 Jul 22;3:56. doi: 10.3389/fvets.2016.00056 (PMC4956649; doi:10.3389/fvets.2016.00056)
Supplement: Supplementary file 1 [file Data_Sheet1.PDF]

Evaluation of HypoThermosol® FRS and Cryostor® as carrier solutions for chilled and frozen equine CB-MSC

Rationale

HypoThermosol® FRS (HTS-FRS) and Cryostor® (CS) are serum and protein-free, commercially available, defined media that have been reported to maintain cell viability better than many other media available during short term hypothermic or cryogenic storage. Equine mesenchymal stromal cells (MSC) are commonly transported chilled or frozen, in serum containing media to veterinary clinics, prior to administration as treatment for a variety of orthopedic injuries. Provided that HTS-FRS or CS do not adversely effect MSC viability, they may be a possible serum free transport media option.

Hypothesis

Simulated transport of chilled or frozen equine MSC suspended in HTS-FRS or CS, respectively, will not result in reduced viability compared to other commonly used media.

Objective

To compare the viability of equine MSC transported chilled or frozen in HTS-FRS or CS, respectively to other commonly used transport media.

Introduction

The in vivo use of cells from fetal bovine serum (FBS)-containing mesenchymal stromal cell (MSC) culture media and cryopreservation media pose risk of immune reactions due to possible

introduction of xenogeneic compounds. Long-term MSC storage in simple crystalline solutions such as saline is associated with reduced cell viability.

CryoStor® (CS) and HypoThermosol® FRS (HTS-FRS) are serum and protein-free cryopreservation and hypothermic preservation media produced by BioLife Solutions Inc. (Bothell, WA, USA). Both products have been adopted by a number of regenerative medicine research laboratories and companies working in the realm of human medicine [1-3]. Both products have been safety tested in animals and humans for so-called 'excipient' use, e.g. used in process and administered to patient along with active ingredient [4]. Such excipient use is in contrast to application in an 'ancillary' manner in which the cell suspension media is removed or washed out prior to cell injection.

In the United States, the products are supported by Type II Master Files (MFs) at the U.S. Food and Drug Administration (FDA). For customers outside the US, BioLife Solutions Inc. works directly with regulatory agencies to help qualify the use of their products in clinical applications. With CS and HTS-FRS being used in ~200 clinical applications worldwide (as of Dec 2015), regulatory classification should not be a barrier for use of the products in veterinary cell-based applications

CryoStor® (CS) contains DMSO at 2, 5 and 10%. These different products are referred to as CS2, CS5 and CS10, respectively. A number of *in vitro* studies with various cell types from different animals have shown that CS is superior or equal to conventional cryomedia containing serum, proteins and DMSO [1, 2, 5]. Excipient use of CS is adapted as a cell-delivery strategy by numerous human regenerative medicine companies, see table at BioLife Solutions website (http://biolifesolutions.com/cryostorclinicaluse/).

HypoThermosol® FRS (HTS-FRS) is a hypothermic preservation media engineered for extended preservation of cells, tissues and organs at 2-8°C. Similarly to CS, HTS-FRS has been adopted as

an excipient medium by a number of regenerative medicine companies working in the realm of human medicine. In vitro studies have shown extended cell viability and live cell recovery using HTS-FRS compared to conventional cell carrier solutions such as physiologic saline and phosphate buffered saline [5]. Excipient use of HTS-FRS for delivery of various human-derived cells to human patients in clinical trials have not been associated with adverse effects related to HTS-FRS [6, 7] and is being pursued by a number of companies, see table at BioLife Solutions website (http://biolifesolutions.com/hypothermosolclinicaluse/).

Materials and Methods

CS studies

Three BM-MSC cultures from independent donors were included in the study (N = 3). The BM-MSC were cryopreserved for 3 weeks in four different cryomedia: CS2, CS5, CS10 and MSC expansion medium containing 10% DMSO (EX10). From each of the 3 animals 5 cryovials were thawed and evaluated. Cell count and viability were determined in duplicate samples using an automated cell counter (NucleoCounter-100, Mandel Scientific, Guelph, ON).

HTS-FRS studies

CB-MSC from 3 independent donors were included (N = 3). The expanded MSCs were separated into 3x 5 million cell aliquots and resuspended in the various cell carrier media and stored at 4 degrees Celsius for either 24 or 48-hour. Following incubation cell number and viability were determined in duplicate samples using an automated cell counter (NucleoCounter-100, Mandel Scientific, Guelph, ON). HTS-FRS was compared to the following MSC carrier suspensions: acellular bone marrow aspirate (ABM), fetal bovine serum (FBS), MSC expansion medium

consisting of DMEM containing 30% FBS (Exp Med), DMEM culture medium without additives (DMEM) and phosphate buffered saline (PBS).

Similar to the CB-MSC HTS-FRS experiment described above, HTS-FRS's ability to support BM-MSC was assessed. A selected number of groups was compared: ABM, FBS, HTS-FRS and regular expansion media consisting of DMEM containing 10% FBS (Media). Three BM-MSC cultures from independent donors were included in the study (N = 3). The thawed and culture expanded MSCs were separated into 3x 5 million cell aliquots and resuspended in the various cell carrier media and stored at 4 degrees Celsius for either 24 or 48-hour. Following incubation cell number and viability were determined in duplicate samples using an automated cell counter (NucleoCounter-100, Mandel Scientific, Guelph, ON).

Results

Both equine BM-MSC and CB-MSC were used to evaluated the performance of HTS-FRS. No significant differences in post-chilled-storage viability (p=0.06) were observed between BM and CB derived MSC cultures. Overall viability of MSC following 24 was greater than 48h chilled storage viability (p<0.001) but no differences in viability were observed between time points for any specific transport media and time combination (24h-94%, 48h-88%, p=0.12), hence transport time was excluded from further analysis. Viability of MSC suspended in HTS-FRS was greater than MSC suspended in ABM (p<0.001), similar to expansion media (p=0.24), and less than MSC suspended in FBS (p=0.02), figure 1.

Viability of cryopreserved MSC suspended in CS10, CS5, or expansion media plus 10% DMSO were not different between groups ($p>0.21$). Viability of MSC cryopreserved in CS2 was significantly different from the three other treatment groups ($p<0.04$) as shown in Figure 2.

Discussion

The *in vitro* performance of CS and HTS-FRS has been tested using both equine umbilical cord blood (CB) and bone marrow-derived (BM) mesenchymal stem cells (MSC) in the Koch lab.

In summary CS5, CS10 and EX10 maintained MSC viability equally well. CS10 cryopreservation resulted in the greatest (albeit insignificantly different) viability of all the solutions tested, performing equal to our in-house cryomedia. HTS-FRS maintained cell viability better than ABM and preserved cell viability equally well as FBS containing medium. HTS-FRS appears as a viable commerical alternative to FBS-based cell-carrier media.

Figures

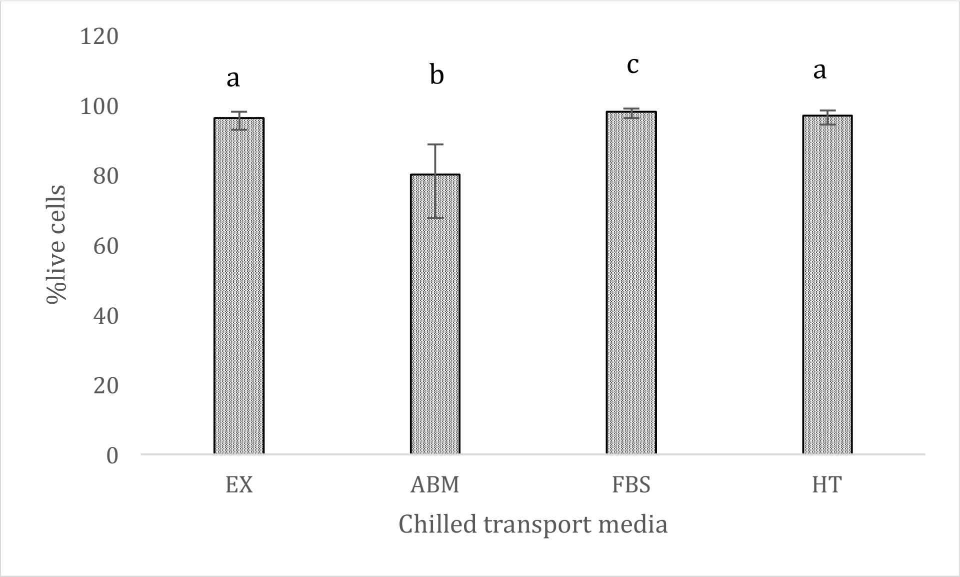

Figure 1: Mesenchymal stromal cell viability following hypothermic (4℃) storage for 24 and 48 hours (data combined) in acellular bone marrow (ABM) DMEM expansion media containing 30% fetal bovine serum (EX), fetal bovine serum (FBS), and HypoThermasol®-FRS (HT). Error bars indicate the 95% confidence interval. Different lowercase letters indicate statistical significance between groups, α=0.05.

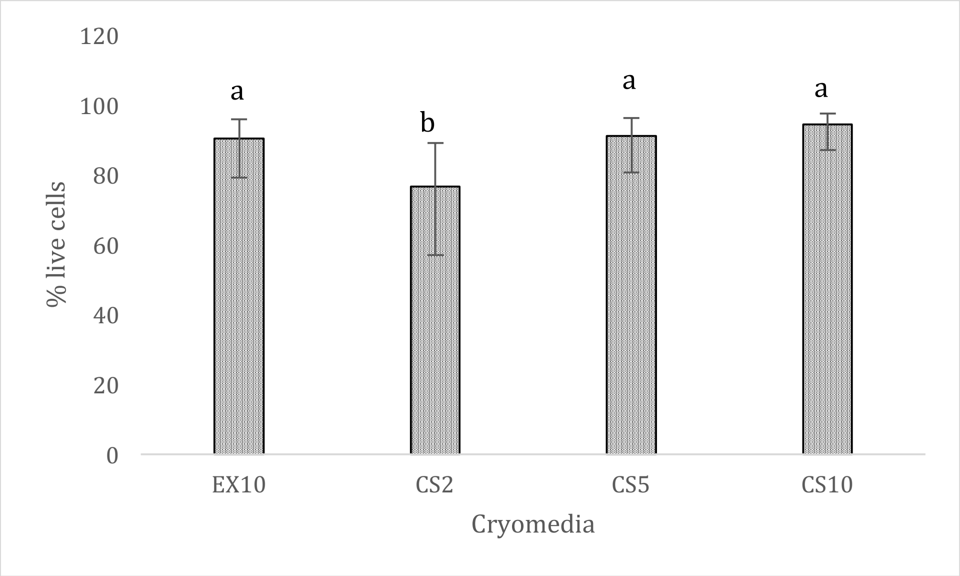

Figure 2.Mesenchymal stromal cell viability following cryopreservation in CryoStor® containing 2, 5, or 10 % DMSO (CS2, CS5, CS10) or serum containing expansion media (DMEM+10% fetal bovine serum)with 10% DMSO added (EX10). Error bars indicate the 95% confidence interval. Different lowercase letters indicate statistical significance between groups, α=0.05.

References

1. Nicoud IB, Clarke DM, Taber G, Stolowski KM, Roberge SE, Song MK, Mathew AJ, Reems JA: Cryopreservation of umbilical cord blood with a novel freezing solution that mimics intracellular ionic composition. *Transfusion* 2012, 52(9):2055-2062.

2. Clarke DM, Yadock DJ, Nicoud IB, Mathew AJ, Heimfeld S: Improved post-thaw recovery of peripheral blood stem/progenitor cells using a novel intracellular-like cryopreservation solution. *Cytotherapy* 2009, 11(4):472-479.

3. Putnam AL, Safinia N, Medvec A, Laszkowska M, Wray M, Mintz MA, Trotta E, Szot GL, Liu W, Lares A *et al*: Clinical grade manufacturing of human alloantigen-reactive regulatory T cells for use in transplantation. *Am J Transplant* 2013, 13(11):3010-3020.

4. Nicoud IB, Nourigat C, Delaney CM: A preclinical safety study of intravenous injection of biopreserved solutions as a vehicle for cellular products. Available at https://www.novabiostorage.com/a-preclinical-safety-study-of-intravenous-injection-of-biopreservation-solutions-as-a-vehicle-for-cellular-products. Accessed January 18, 2016.

5. Ginis I, Grinblat B, Shirvan MH: Evaluation of bone marrow-derived mesenchymal stem cells after cryopreservation and hypothermic storage in clinically safe medium. *Tissue Eng Part C Methods* 2012, 18(6):453-463.

6. Povsic TJ, O'Connor CM, Henry T, Taussig A, Kereiakes DJ, Fortuin FD, Niederman A, Schatz R, Spencer Rt, Owens D *et al*: A double-blind, randomized, controlled, multicenter study to assess the safety and cardiovascular effects of skeletal myoblast implantation by catheter delivery in patients with chronic heart failure after myocardial infarction. *Am Heart J* 2011, 162(4):654-662 e651.

7. Bartunek J, Behfar A, Dolatabadi D, Vanderheyden M, Ostojic M, Dens J, El Nakadi B, Banovic M, Beleslin B, Vrolix M *et al*: Cardiopoietic stem cell therapy in heart failure: the C-CURE (Cardiopoietic stem Cell therapy in heart failURE) multicenter randomized trial with lineage-specified biologics. *J Am Coll Cardiol* 2013, 61(23):2329-2338.
