# Supplementary material for: Response to Intravenous Allogeneic Equine Cord Blood-Derived Mesenchymal Stromal Cells Administered from Chilled or Frozen State in Serum and Protein-Free Media
Source: Front Vet Sci. 2016 Jul 22;3:56. doi: 10.3389/fvets.2016.00056 (PMC4956649; doi:10.3389/fvets.2016.00056)
Supplement: Supplementary file 2 [file Data_Sheet2.PDF]

Intra-articular HypoThermosol® FRS injection does not result in abnormal synovial fluid routine cytology parameters in vivo.

Rationale

HypoThermosol® FRS (HTS-FRS) is a serum-free and protein-free, commercially available, defined media that has been reported to maintain cell viability better than many other media available during short term hypothermic storage. HTS-FRS has been shown to maintain human mesenchymal stromal cell (MSC) viability better than many other available media. Data originating from our research group indicates equine cord blood (CB) MSC maintain higher viability when stored in HTS-FRS compared to phosphate buffered saline (PBS) and acellular bone marrow aspirate. This data also indicates that HTS-FRS performed similarly to serum containing media in preserving CB-MSC viability for short-term hypothermic storage (supplemental file 1). As such HTS-FRS appears to be an ideal carrier solution for transportation of CB-MSC suspensions provided it does not cause adverse reactions when injected in vivo in an excipient manner.

Hypothesis

Intra-articular injection of HTS-FRS does not result in changes in synovial fluid routine cytology values

Objective

To compare the change in synovial fluid routine cytology parameters following intra-articular injection of either HTS-FRS or PBS.

Introduction:

Intra-articular injections are commonly performed in the horse for treatment of joint injuries. In addition to conventional therapies for joint injury intra-articular injection of MSC suspensions is becoming common as a treatment for joint injury.

MSC suspensions require transport from laboratory to veterinary clinic, usually by commercial overnight carrier. Decreased MSC viability has been demonstrated following transportation in various media (1, 2). One strategy reported to slow the decrease in MSC viability is to transport MSC in serum containing media. If the serum is allogeneic or xenogeneic in nature, then it is often recommended that the MSC undergo multiple washes prior to injection to remove the majority of foreign antigens introduced by the serum. This process is inconvenient, associated a decrease of total MSC numbers and increases the risk of bacterial contamination. A cell carrier media which requires removal as described prior to injection of the MSC is referred to as an ancillary media. Excipient media is a cell carrier solution which provides necessary support but is otherwise unreactive and can be injected with the MSC. Excipient media allows a convenient and standardized final product formulation since no manipulations are required at the time of treatment. HTS-FRS is a cell preservation medium for excipient use with chilled mammalian cells. HTS-FRS is serum-free and protein-free, commercially available, procured with adherence to current Good Manufacturing Practices (GMP) and is optimized for maintaining cell viability at 2-8°C. HTS-FRS has been shown to preserve viability of cells, tissues, or organs better than cells, tissues or organs transported in many other media (3-8). We hypothesized that synovial fluid total nucleated cell count (TNCC), total protein (TP), and differential cell count would not be different between treatment groups following a single intra-articular injection of HTS-FRS or PBS.

Materials and Methods

Research animals

Three mature healthy male Standardbred horses that had not received any medications for at least two months were selected for use in this study. Each animal received a general physical exam prior to commencement and at 8, 24, 48, and 72 hours post-injection. Horses were examined at distance for the two weeks following the last synovial fluid sampling to evaluate for obvious lameness that may indicate adverse reaction or infection. All procedures complied with institutional animal care committee protocols approved for this study (Koenig, University of Guelph Animal Care Protocol # 1879).

Within the same horse, each animal's tarsocrural (TC) joints were randomly assigned to HTS-FRS or PBS treatment groups by coin flip. Each horse was sedated (detomidine hydrochloride 0.01 mg/kg, butorphanol tartarate 0.01 mg/kg—i.v.) prior to injection. Both hocks were clipped and prepared for aseptic injection into the tarsocrural (TC) joint. Arthrocentesis was performed using a 20 Ga x 1" hypodermic needle, and 2 mL of synovial fluid was collected. Following this, 2mL HTS-FRS was injected prior to removal of the needle. The contralateral TC joint underwent a similar procedure but received 2 mL of PBS instead of HTS-FRS. Subsequently, at 8, 24, 48, and 72 hours post injection, 2 mL of SF was collected. Each SF sample was injected into glass EDTA containing vials and immediately transported to the laboratory at room temperature. Each vial was submitted to our university's veterinary diagnostic laboratory for routine cytological analysis, which included total nucleated cell count (TNCC) (Coulter Z2 nucleated cell counter, Beckman Coulter, Hialeah FL), total protein (TP) by refractometry, and differential cell count (blinded board-certified clinical pathologist). Published reference ranges were used for interpretation of results (9).

Statistical analysis

Raw data was imported into statistical software (SAS 9.2, SAS institute, Carey NC). Data were analyzed using a general linear mixed model using the PROC MIXED function. Residual analysis was used to determine if ANOVA assumptions were met, to detect outliers, and to evaluate the need for data transformation. When comparisons were made between simple effects, the simple effect ANOVA tables were used to determine statistical significance provided $p<0.05$ in for the overall ANOVA. For the purpose of determining statistical significance, α was set at 0.05.

Results

Throughout the duration of this study all horses maintained physical exam parameters within established normal ranges and did not exhibit lameness at any time.

Significant differences were not detected between HTS-FRS and PBS treated joints at any time point in TNCC, TP, and the percentage of neutrophils observed in the synovial fluid (figures 1-3).

A significant time effect was observed in the TNCC ($p=0.0006$) and percentage of neutrophils in the synovial fluid ($p<0.0001$). Compared to baseline samples significant increases in TNCC were observed at 24, 48, and 72 hours post injection, $p=0.0004$, 0.0001, 0.002, respectively (figure 1). Compared to baseline samples significant increases in the percent of neutrophils were observed at all time-points post injection, $p<0.0001$ each (figure 3).

Discussion

We report no significant differences the TNCC, TP, and percentage of neutrophils in the synovial fluid following injection of either HTS-FRS or PBS into the TC joint. These findings are of significance as they indicate that HTS-FRS does not induce a detectable adverse reaction when injected intra-articularly into equine joints.

Significant increases in TNCC and the percentage of neutrophils within the synovial fluid were observed compared to baseline levels. These changes represent the result of inflammation induced by the injection process and the result of mild intra-articular hemorrhage following injection. Despite these changes TNCC and TP remained within normal ranges (9) throughout the duration of the study. The percentage of neutrophils in the synovial fluid increased beyond what was expected for healthy joints injected with a balanced electrolyte solution. In these instances, when evaluating the synovial fluid cytospin slides, the clinical pathologist's report indicated varying degrees of hemorrhage in conjunction with elevated neutrophil proportions. As such, we conclude that the neutrophil populations observed occurred as a result of iatrogenic hemorrhage that occurred during synovial fluid collection.

We conclude that HTS-FRS does not induce an inflammatory response beyond that which is associated with arthrocentesis and injection of a balanced electrolyte solution. As such we consider HTS-FRS safe for injection into equine joints.

Acknowledgements:

We would like to thank BioLife Solutions®, Bothell, WA, for supplying HypoThermosol® and CryoStor® for use in this study free of charge. Funding for this study was generously provided by Equine Guelph.

Figures

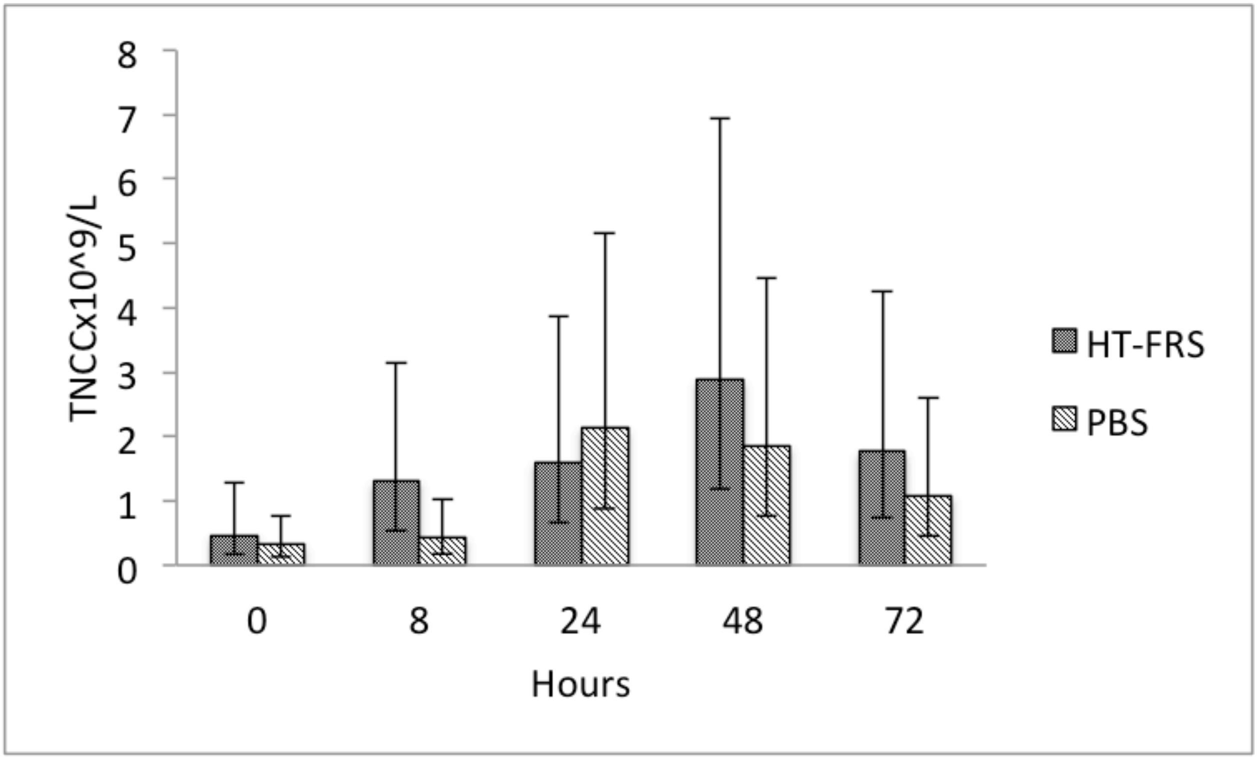

Figure 1: Total nucleated cell count in synovial fluid following injection of HypoThermosol® FRS (HTS-FRS) into one tarsalcrural joint with phosphate buffered saline (PBS) control. Significant differences were not detected between joints receiving HTS-FRS and PBS at any time point. Error bars represent 95% CI (n=3).

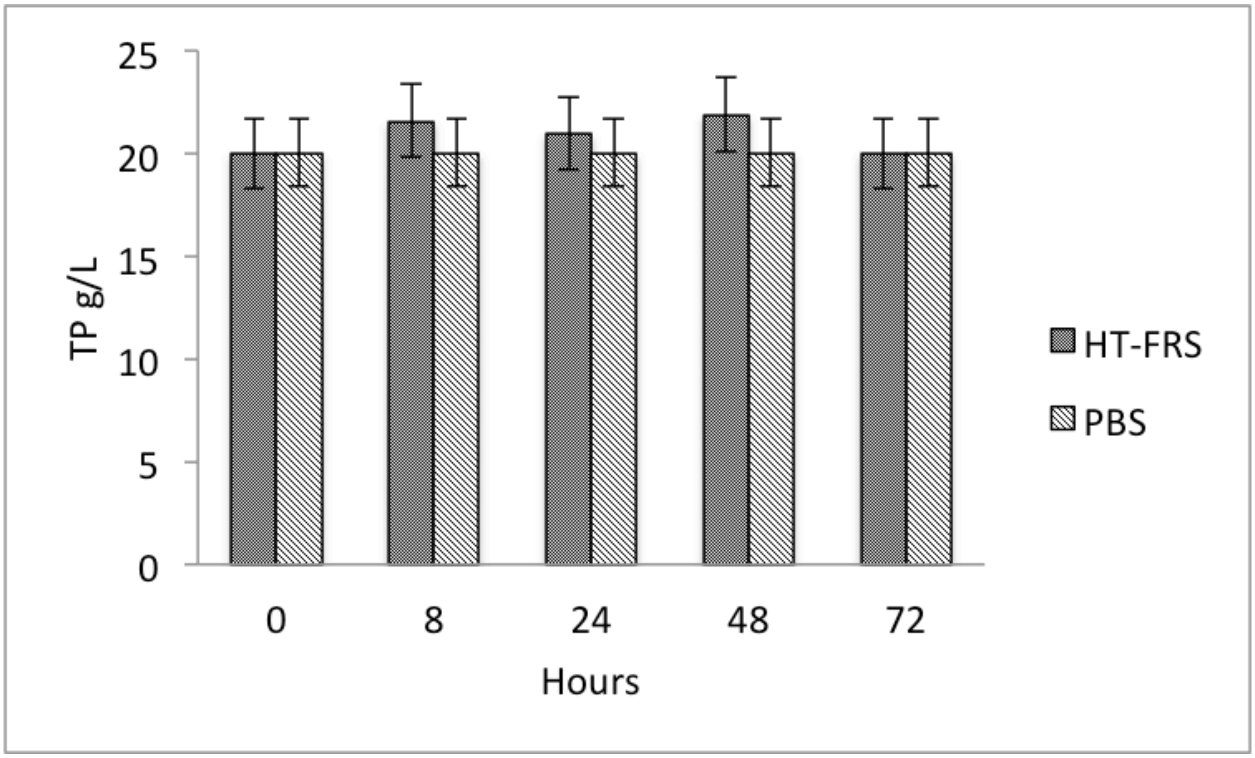

Figure 2: Total protein in synovial fluid following injection of HypoThermosol® FRS (HTS-FRS) into one tarsalcrural joint with phosphate buffered saline (PBS) control. Significant differences were not detected between joints receiving HTS-FRS and PBS at any time point. Error bars represent 95% CI (n=3).

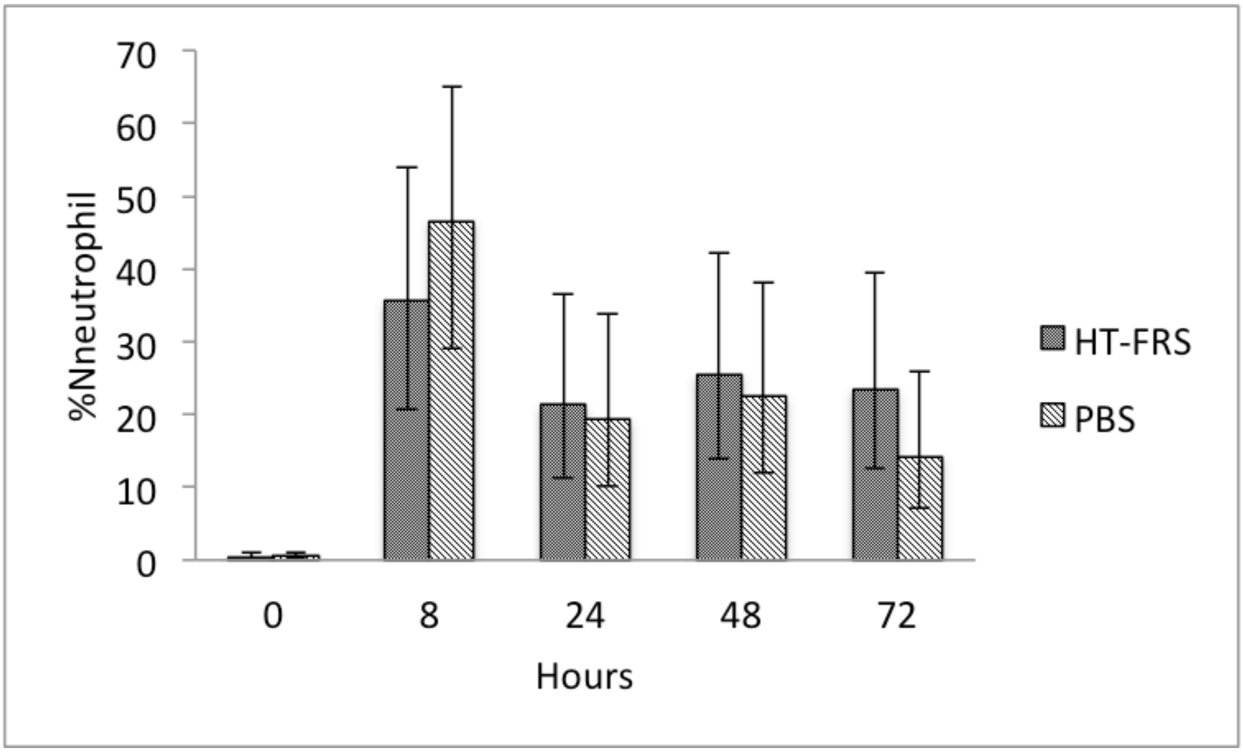

Figure 3: Differential cell count reported as neutrophil or mononuclear cell populations in synovial fluid following injection of HypoThermosol® FRS (HTS-FRS) into one tarsalcrural joint with phosphate buffered saline (PBS) control. Significant differences were not detected between joints receiving HTS-FRS and PBS at any time point. Error bars represent 95% CI (n=3)

References

1. Nikolaev NI, Liu Y, Hussein H, Williams DJ. The sensitivity of human mesenchymal stem cells to vibration and cold storage conditions representative of cold transportation. J R Soc Interface. 2012 Oct 7;9(75):2503-15.

2. Garvican ER, Cree S, Bull L, Smith RK, Dudhia J. Viability of equine mesenchymal stem cells during transport and implantation. Stem Cell Res Ther. 2014 Aug 8;5(4):94.

3. Baust JM, Van Buskirk R, Baust JG. Modulation of the cryopreservation cap: Elevated survival with reduced dimethyl sulfoxide concentration. Cryobiology. 2002 Oct;45(2):97-108.

4. Bessems M, Doorschodt BM, van Vliet AK, van Gulik TM. Preservation of rat livers by cold storage: A comparison between the university of wisconsin solution and hypothermosol. Ann Transplant. 2004;9(2):35-7.

5. Cook JR, Eichelberger H, Robert S, Rauch J, Baust JG, Taylor MJ, et al. Cold-storage of synthetic human epidermis in HypoThermosol. Tissue Eng. 1995 Winter;1(4):361-77.

6. Dahdah NS, Taylor MJ, Russo P, Wagerle LC. Effects of hypothermosol, an experimental acellular solution for tissue preservation and cardiopulmonary bypass, on isolated newborn lamb coronary vessels subjected to ultra profound hypothermia and anoxia. Cryobiology. 1999 Aug;39(1):58-68.

7. Ginis I, Grinblat B, Shirvan MH. Evaluation of bone marrow-derived mesenchymal stem cells after cryopreservation and hypothermic storage in clinically safe medium. Tissue Eng Part C Methods. 2012 Jun;18(6):453-63.

8. Mathew AJ, Baust JM, Van Buskirk RG, Baust JG. Cell preservation in reparative and regenerative medicine: Evolution of individualized solution composition. Tissue Eng. 2004 Nov-Dec;10(11-12):1662-71.

9. Caron J. Osteoarthritis. In: Ross M, Dyson S, editors. In: Diagnosis and Management of Lameness in the Horse. St. Louis: Saunders; 2003. p. 572-591.
