# Supplementary material for: Response to Intravenous Allogeneic Equine Cord Blood-Derived Mesenchymal Stromal Cells Administered from Chilled or Frozen State in Serum and Protein-Free Media
Source: Front Vet Sci. 2016 Jul 22;3:56. doi: 10.3389/fvets.2016.00056 (PMC4956649; doi:10.3389/fvets.2016.00056)
Supplement: Supplementary file 3 [file Data_Sheet3.PDF]

Additional figures comparing hematological, biochemical, coagulation, and lymphocyte subsets of ponies receiving a 10 mL IV bolus of either HTS-FRS, CS-10, or saline and pre-injection MSC viability

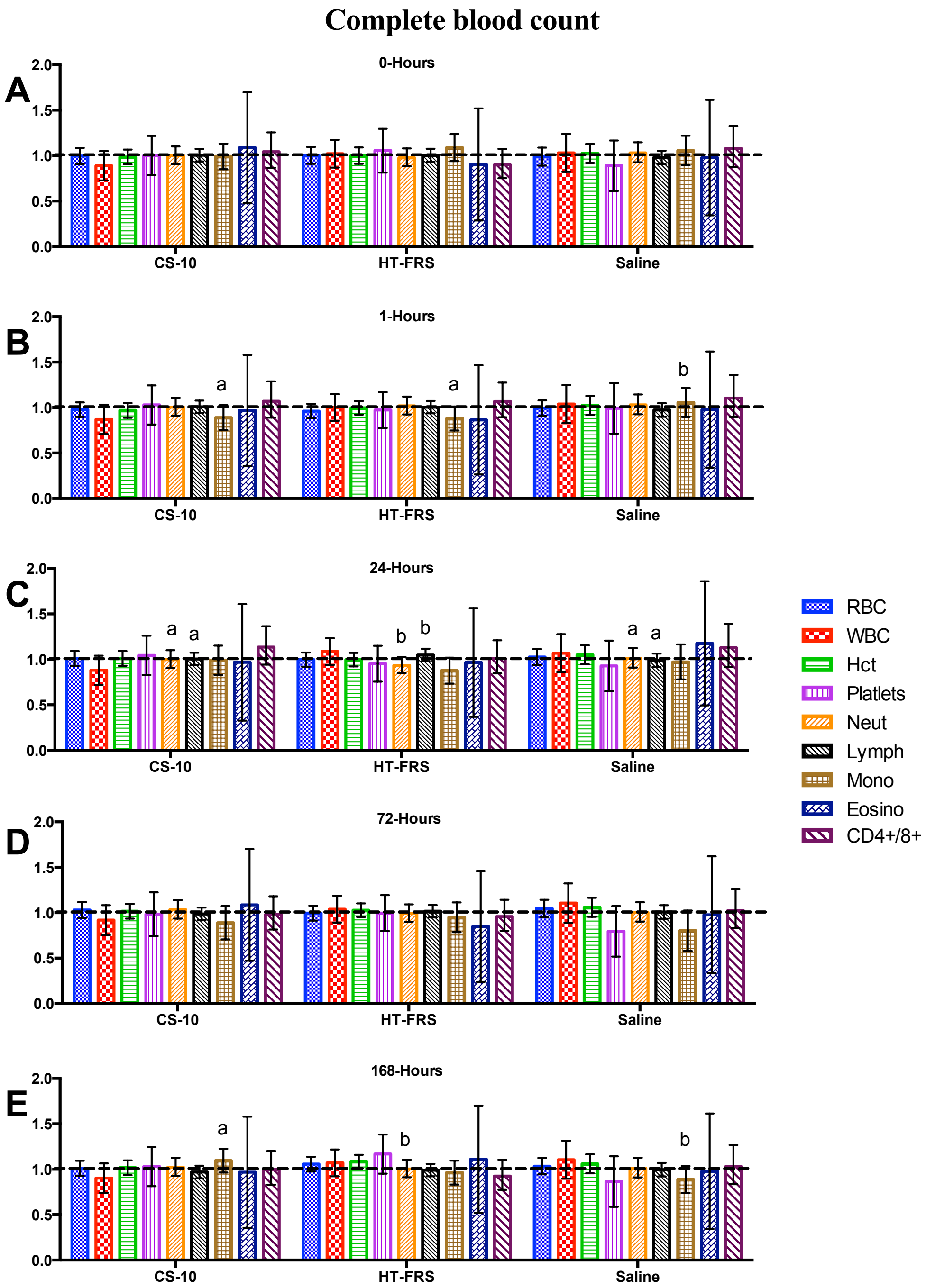

Figure A: Percent difference from pre-injection measurements (black dotted line) of complete blood count A) 0 h, B) 1 h, C) 24 h D) 72 h, E) 168 h following intravenous injection of 10mL HypoThermosol® (HTS-FRS), CryoStor® CS10 (CS10) or Saline (n=3 each group). Error bars represent 95% confidence interval. Different letters indicate statistical differences between treatment and control groups for a specific parameter.

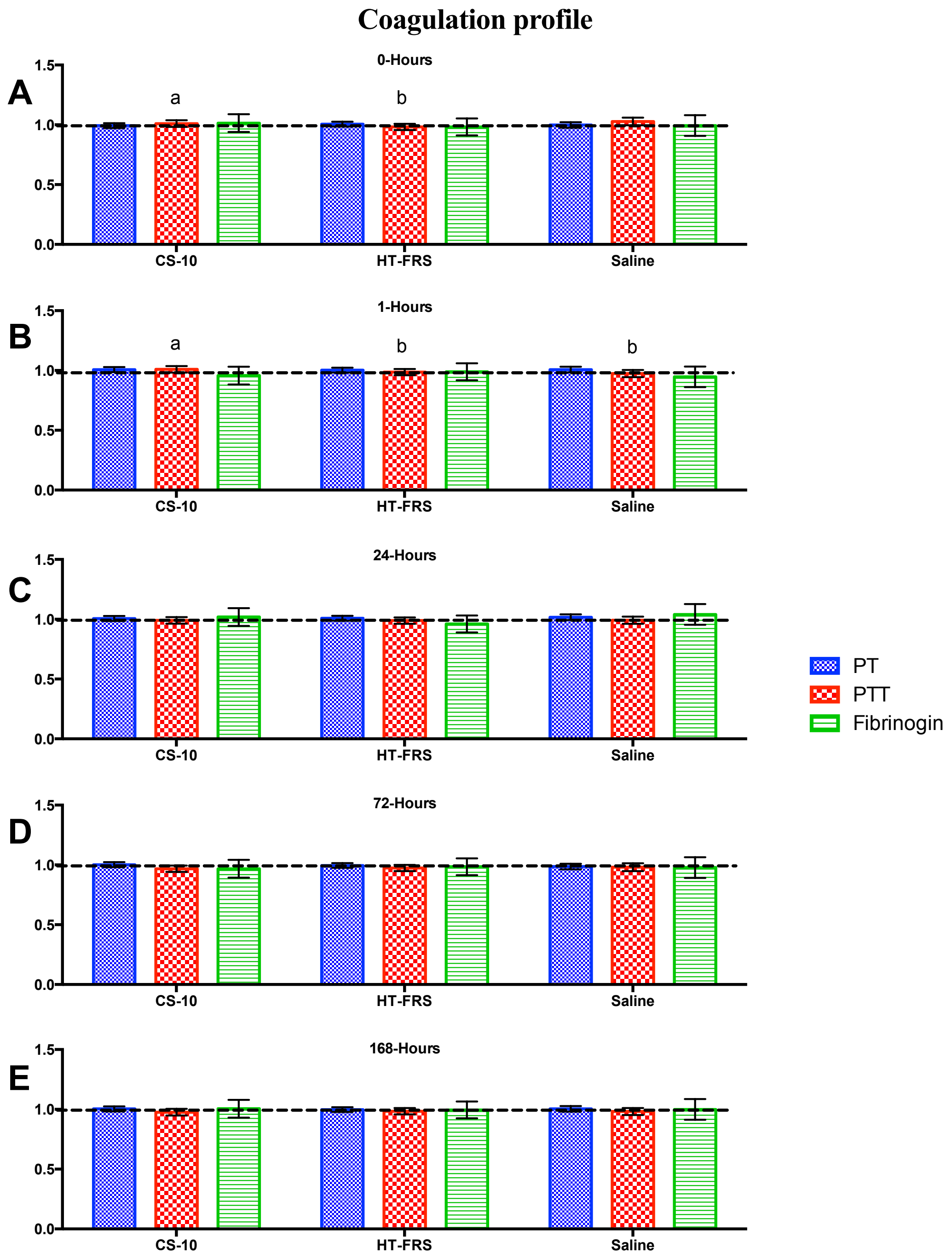

Figure B: Percent difference from pre-injection measurements (black dotted line) coagulation profile A) 0 h, B) 1 h, C) 24 h D) 72 h, E) 168 h following intravenous injection of 10mL HypoThermosol® (HTS-FRS), CryoStor® CS10 (CS10) or Saline (n=3 each group). Error bars represent 95% confidence interval. Different letters indicate statistical differences between treatment and control groups for a specific parameter.

Biochemistry Profile

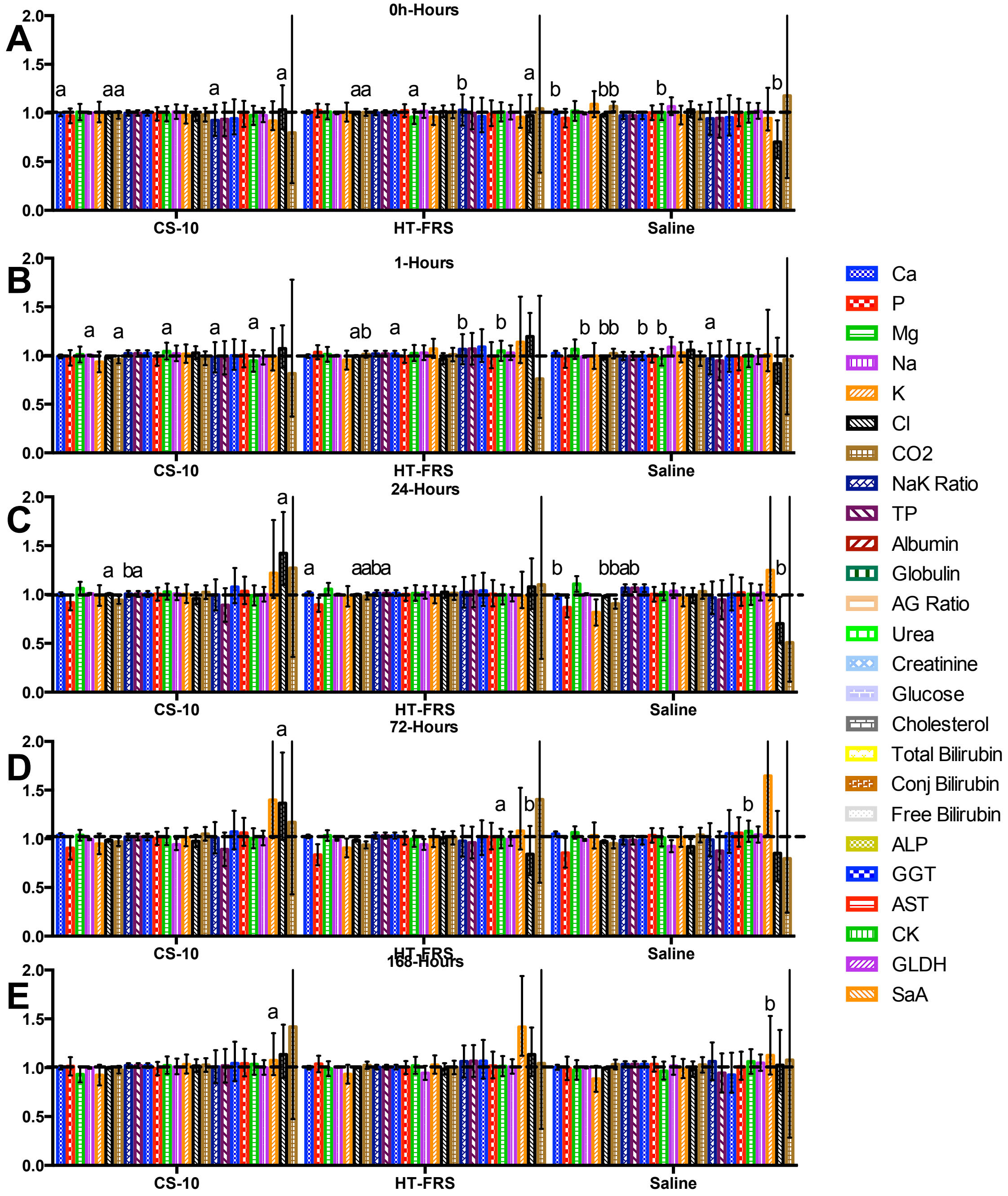

Figure C: Percent difference from pre-injection measurements (black dotted line) of biochemistry profile A) 0 h, B) 1 h, C) 24 h D) 72 h, E) 168 h following intravenous injection of 10mL HypoThermosol® (HTS-FRS), CryoStor® CS10 (CS10) or Saline (n=3 each group). Error bars represent 95% confidence interval. Different letters indicate statistical differences between treatment and control groups for a specific parameter.

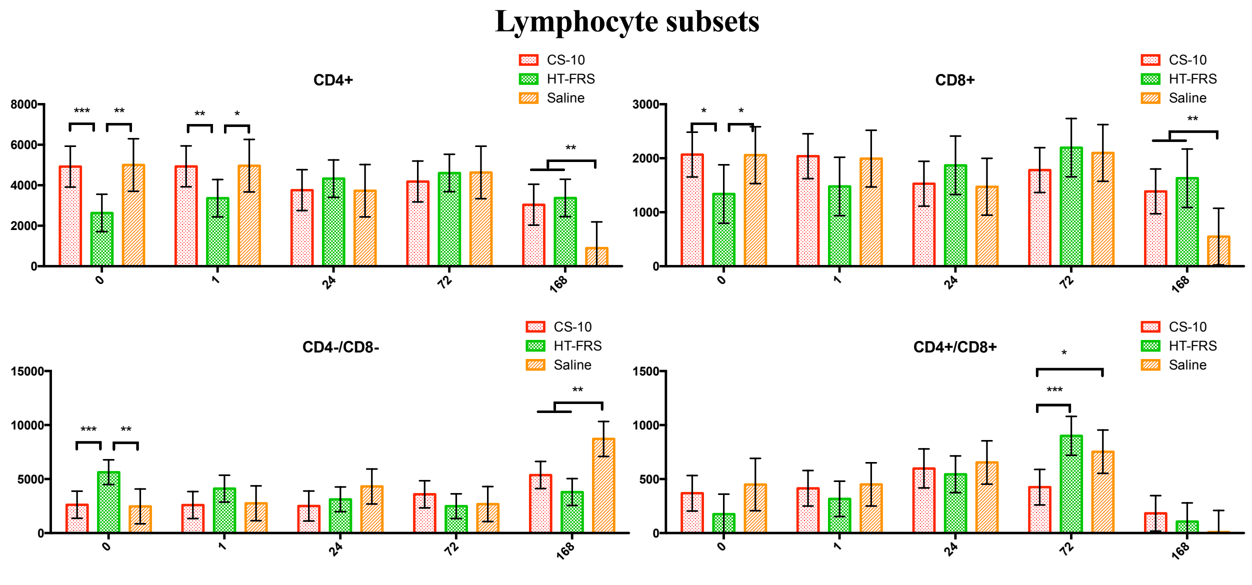

Figure D: CD4+, CD8+, CD4+/CD8+ (double positive), and CD4-/CD8- (double negative) lymphocyte populations 10mL HypoThermosol® (HTS-FRS), CryoStor® CS10 (CS10) or Saline (n=3 each group). *p<0.05, **p<0.01, ***p<0.001

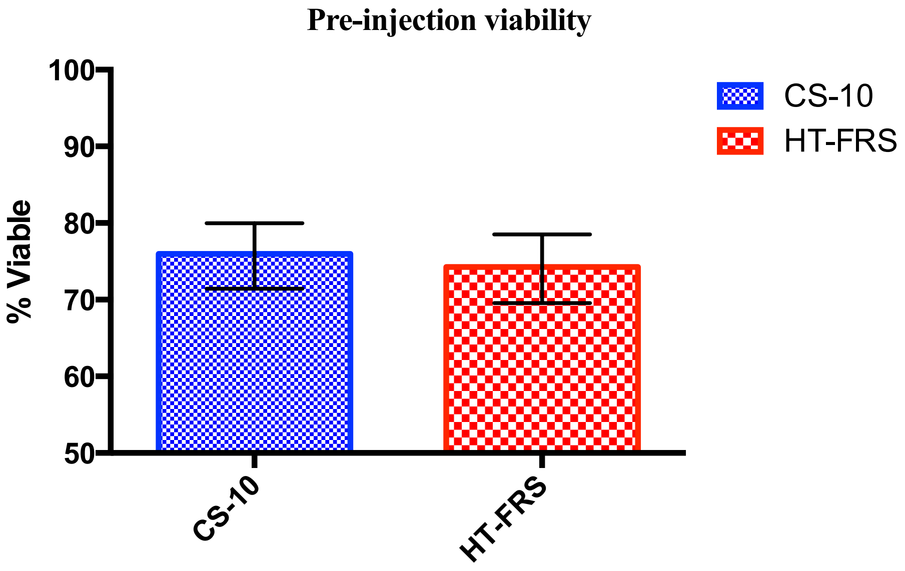

Figure E: Viability of residual CB-MSC suspension following simulated transport prior to injection. $5x10^7$ pooled allogeneic CB-MSC derived from 5 horses suspended in either HypoThermosol® FRS (HTS-FRS) or CryoStor® CS10 (CS10) and stored for 24h under simulated transport conditions (2-8°C passive cooling shipping container in the case of CB-MSC suspended in HTS-FRS, dry ice cooler in the case of CB-MSC suspended in CS10). *p<0.05, **p<0.01, ***p<0.001
